# Deuteron Chemical Exchange Saturation Transfer for the Detection of Slow Motions in Rotating Solids

**DOI:** 10.3389/fmolb.2021.705572

**Published:** 2021-07-27

**Authors:** Liliya Vugmeyster, Dmitry Ostrovsky, Alexander Greenwood, Riqiang Fu

**Affiliations:** ^1^ Department of Chemistry, University of Colorado Denver, Denver, CO, United States; ^2^ Department of Mathematics, University of Colorado Denver, Denver, CO, United States; ^3^ Department of Chemistry, University of Cincinnati, Cincinnati, OH, United States; ^4^ National High Field Magnetic Laboratory, Tallahassee, FL, United States

**Keywords:** CEST (chemical exchange dependent saturation transfer), deuterium, solids-state NMR, biomolecular dynamics, conformational exchange

## Abstract

We utilized the ^2^H Chemical Exchange Saturation Transfer (CEST) technique under magic angle spinning (MAS) conditions to demonstrate the feasibility of the method for studies of slow motions in the solid state. For the quadrupolar anisotropic interaction, the essence of CEST is to scan the saturation pattern over a range of offsets corresponding to the entire spectral region(s) for all conformational states involved, which translates into a range of −60–+ 60 kHz for methyl groups. Rotary resonances occur when the offsets are at half-and full-integer of the MAS rates. The choice of the optimal MAS rate is governed by the condition to reduce the number of rotary resonances in the CEST profile patterns and retain a sufficiently large quadrupolar interaction active under MAS to maintain sensitivity to motions. As examples, we applied this technique to a well-known model compound dimethyl-sulfone (DMS) as well as amyloid-β fibrils selectively deuterated at a single methyl group of A2 belonging to the disordered domain. It is demonstrated that the obtained exchange rate between the two rotameric states of DMS at elevated temperatures fell within known ranges and the fitted model parameters for the fibrils agree well with the previously obtained value using static ^2^H NMR techniques. Additionally, for the fibrils we have observed characteristic broadening of rotary resonances in the presence of conformational exchange, which provides implications for model selection and refinement. This work sets the stage for future potential extensions of the ^2^H CEST under MAS technique to multiple-labeled samples in small molecules and proteins.

## Introduction

Chemical Exchange Saturation Transfer (CEST) experiments provide insights into the molecular dynamics in solution and solid-state NMR studies (Siemer et al., 2010; Bouvignies and Kay, 2012; Vallurupalli et al., 2012; Palmer, 2014; Rovó and Linser, 2018; Palmer and Koss, 2019). They employ weak RF fields for the saturation of selected frequencies as a function of resonance offsets. In most cases, the fluctuations of the isotropic chemical shift interaction is probed when the conformational states have inequivalent chemical shifts. However, anisotropic interactions can also be targeted in the solid state. These measurements are expected to be useful for probing molecular dynamics in a variety of biological systems, including protein fibrils, aggregates and microcrystals. They can elucidate the presence of minor conformational states exchanging with the major state at a slow timescale with the rate constant in the 5⋅10^2^–5⋅10^6^ s^−1^ range, with the highest sensitivity around 1⋅10^4^–5⋅10^5^ s^−1^. Local motional modes of protein side chains, such as rotameric exchange of methyl-bearing side chains, as well as aromatic ring flips can also be probed with the use of this technique. In addition, backbone motions of C_α_ deuterons can be elucidated for mobile sites such as loop regions.

We recently demonstrated the effectiveness of the technique for fluctuations of the anisotropic quadrupolar tensor of ^2^H nuclei under static conditions in the solid state (Vugmeyster et al., 2020). The goal of this work is to expand the methodology for magic angle spinning (MAS) conditions. MAS has proven to be indispensable for dynamic studies of many biomolecular samples with multiple labels (Krushelnitsky et al., 2014; van der Wel, 2017; Rovó, 2020). In this work, we use a single-labeled sample to demonstrate the effectiveness of the ^2^H CEST experiment, which encourages follow-up studies employing polarization transfer approaches to achieve site-specific resolution (Grey et al., 1993; Bjerring et al., 2012; Akbey et al., 2014; Jain et al., 2014; Matlahov and van der Wel, 2018).

In particular, we apply the experiment to the model compound dimethyl-sulfone (DMS) deuterated at its two methyl groups that undergo 2-site rotameric exchange (Frydman et al., 1994; Brown et al., 1996; Gerardy-Montouillout et al., 1996; Favre et al., 1998; Quinn and McDermott, 2012) and to amyloid-β fibrils (Aβ_1-40_) with the deuterium label at a mobile methyl group of the A2 residue belonging to the disordered N-terminal domain, for which we previously determined the dynamics using static ^2^H solid-state NMR techniques (Au et al., 2019; Vugmeyster et al., 2019; Vugmeyster et al., 2020). The experimental work is complemented with theoretical considerations using the Liouvillian formalism (Bain and Berno, 2011) and insights into the main features of the CEST profiles resulting from simple 2-site exchange simulations. Our combined experimental and theoretical/modeling analysis allows us to outline consideration for optimization of the technique and define the ranges of its sensitivity to motions.

## Experimental

### Materials

DMS-D_6_ and hexamethyl-benzene-D_18_ were purchased from Cambridge Isotope Laboratories, Inc. (MA) and packed as a powder into rotors. The Aβ_1-40_ fibrils labeled at the A2-CD_3_ site were prepared as previously described in the 3-fold symmetric toxic polymorph (Au et al., 2019; Vugmeyster et al., 2020). The monomeric sequence of the Aβ_1-40_ peptide is D(**A-CD**_**3**_ **)**EFRHDSGYEVHHQKLVFFAEDVGSNKGAIIGLMVGGVV. The lyophilized powder was hydrated to 200% by weight with deuterium-depleted water using direct pipetting and equilibrating at room temperature for 5 days. The hydrated sample was then packed into a 2.5 mm rotor.

### NMR Spectroscopy

The measurements for 10 and 25 kHz MAS frequency were performed at 17.6 T Bruker Avance I spectrometer equipped with a Bruker 2.5 mm HXY probe. The measurements at 60 kHz MAS were performed at 14.1 T Bruker neo console spectrometer equipped with a Bruker 1.3 mm HXY probe. The high-power 90° RF pulses corresponded to 2 μs The number of scans for the acquisition was between 32 and 64 for DMS and between 2048 and 3072 for the protein sample. The inter-scan delay was set between 0.5 and 2 s. Because one potential source of systematic error in the ^2^H CEST intensities is probe detuning, data collection is optimized when the order of the offsets is randomized.

DMS longitudinal relaxation times (*T*_1_) are very sensitive to temperatures in around 40–85°C range, and thus we have used it as an internal calibrations standard to obtain the actual temperature in the samples (Vugmeyster and Ostrovsky, 2019). For the 60 kHz MAS rate, the effect of MAS on *T*_1_ for a given methyl 3-site jump rate was simulated. We also confirmed that the weak-amplitude RF field does not contribute to heating with the chosen relaxation delay value. The longitudinal relaxation times were measured using the inversion recovery experiment, which included a heat compensation block to match the temperature conditions of the sample in the CEST measurements.

### Modeling

The simulations were performed on a cluster comprising six x86_64 computer nodes**.** Each node had 16 Intel Xenon Silver dual core CPUs and 16 GB of memory. The procedures closely followed those developed for static conditions in prior work (Vugmeyster et al., 2020). Here, we focus on the details pertinent to MAS conditions.

The evolution of the coherences under MAS was modeled by the direct numerical integration of the Liouville–von Neumann equation (Supplementary Equation. S1; all the notations used are elaborated in the Theory section of the Supplementary Material SI1). The coherent time-dependent values of the quadrupolar frequency $\omega_{Q}\left(t\right)$ for each site are given by Eq. 4. The numerical integration for each saturation time delay was separated into two blocks. The first block comprised the calculation of the evolution matrix for a single MAS rotation $T\text{exp}\left(\overset{2\pi /\omega_{MAS}}{\underset{0}{\int }}Ldt\right)$, where *T* stands for the time-ordered exponential function and *L* is the Liouvillian operator of Supplementary Equation. S1. This integration was performed by numerical quadrature with 20 time steps along a single MAS rotation period $2\pi /\omega_{MAS}$. For an individual step, the exponentiation was conducted with fixed $\omega_{Q}\left(t\right)$ values using the internal MATLAB function (Higham, 2005; Al-Mohy and Higham, 2010). We did not use the approximation involving separate integration steps due to the coherent evolution and exchange processes (Saalwächter and Fischbach, 2002). The sufficiency of 20 time steps was confirmed by comparing the results with selected trials with 100 steps. The high consistency of the results holds down to values of $\omega_{MAS}/2\pi$ as low as 1 kHz. The second part of the calculation of the saturation period evolution involved taking the appropriate powers of the evolution matrix for a single MAS rotation as well as the additional multiplicative factor involving the fractional part of the rotation calculated in a similar manner. The equilibrium component for the Zeeman order coherence was introduced phenomenologically as an additional term in the density matrix similar to the Bloch–McConnell treatment of the z-component of magnetization (Vallurupalli et al., 2012).

The detection block was performed starting with the approach described for the static case. As usual, the value of $S_{z}$ for each site was rotated onto the transverse plane and the simulation of the evolution during the acquisition period followed a similar outline, but involved only the transverse coherences. The time step of calculations was selected as the smaller of 1/20 of the MAS period and FID dwell time.

For the fibrils, longitudinal relaxation was taken into account phenomenologically (with *T*_1_ = 50 ms) by including an additional term in the Liouvillian evolution matrix, which was identical for all eight coherences. This approach was tested for DMS for which the inclusion of the 3-site jumps mode explicitly yielded the same results. To model the effects of the RF inhomogeneity on the CEST profiles, we included five values of $\omega_{RF}/2\pi$ in the ±0.5 kHz range from its central value and averaged the simulated profiles.

**FIGURE 1:**
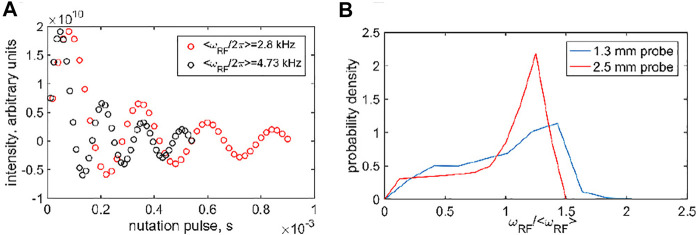
**(A)** Examples of the ^2^H nutation measurements used to determine RF inhomogeneity, shown for the 1.3 mm Bruker HXY probe using the DMS-D_6_ central band intensity at the 60 kHz MAS rate at 14.1 T. **(B)** Resulting inhomogeneity profiles, obtained as the Fourier analysis of the nutation data, as a function of *ω*_RF_/<*ω*_RF_ > obtained for a <*ω*_RF_ > range of 2.8–20 kHz for the 1.3 mm Bruker HX probe using DMS-D_6_ (at 14.1 T) and 5–31 kHz for the 2.5 mm Bruker HXY probe using HMB-D_18_ (at 17.6 T).

The RF inhomogeneity profiles (Figure 1B) were discretized by selecting a grid of either six points (for resonance offset values outside the −2–2 kHz region) or 30 points (in the −2–2 kHz region). A larger number of points for the central region was needed due to the enhanced coherent oscillations. The six grid points of the RF inhomogeneity profiles corresponded to the RF field values at 0.25, 0.5, 0.75, 1, 1.25, and 1.5 multiples of the average frequency with the respective relative weights (0.084, 0.143, 0.126, 0.176, 0.285, 0.187 and 0.065, 0.097, 0.103, 0.236, 0.499, 0) for the 1.3 and 2.5 mm probes, respectively. The 30-point grids were obtained by the interpolation of the six-point grids.

## Results and Discussion

### Details on the Systems and Known Motional Models

**FIGURE 2:**
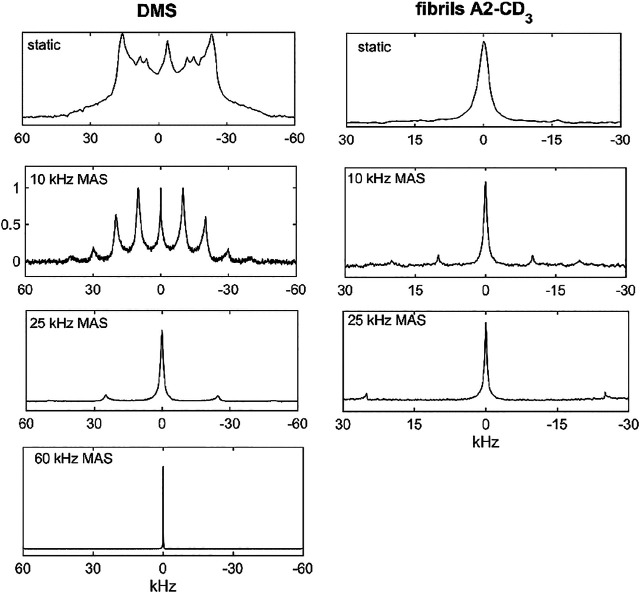
Spectra for DMS-D_6_ (left column) and hydrated Aβ_1-40_ fibrils in the 3-fold symmetric polymorph labeled at the A2-CD_3_ site (right column). The following conditions applied: static data–14.1 T and 76°C for DMS and 37°C for the fibrils; 10 and 25 kHz MAS rates–17.6 T and 76°C for DMS and 37°C for the fibrils; 60 kHz MAS rate for DMS only–14.1 T and 55°C. The number of scans and processing parameters are listed in Supplementary Table S1.

DMS has been widely used as a model system for solid-state NMR technique development and, in particular, for deuteron NMR. Its methyl group undergoes a 2-site rotameric exchange with an angle of rotation of 180° around the C_2_ axis of the molecule (Frydman et al., 1994; Brown et al., 1996; Gerardy-Montouillout et al., 1996; Favre et al., 1998; Quinn and McDermott, 2012). These motions are the most pronounced above around 45°C. We have previously utilized this system to develop a deuteron CEST measurement under static conditions and extend it in this work to MAS conditions (Vugmeyster and Ostrovsky, 2019). The ^2^H spectra under static and MAS conditions (at spinning rates of 10, 25, and 60 kHz) are shown in Figure 2. Although there are some spectral distortions due to the motions in the regime in which the flip rate is of the order of the effective value of the quadrupolar constant, the overall width of the pattern remains largely unchanged by the motions. The quadrupolar coupling constant is 55 kHz after averaging over fast methyl rotations.

**FIGURE 3:**
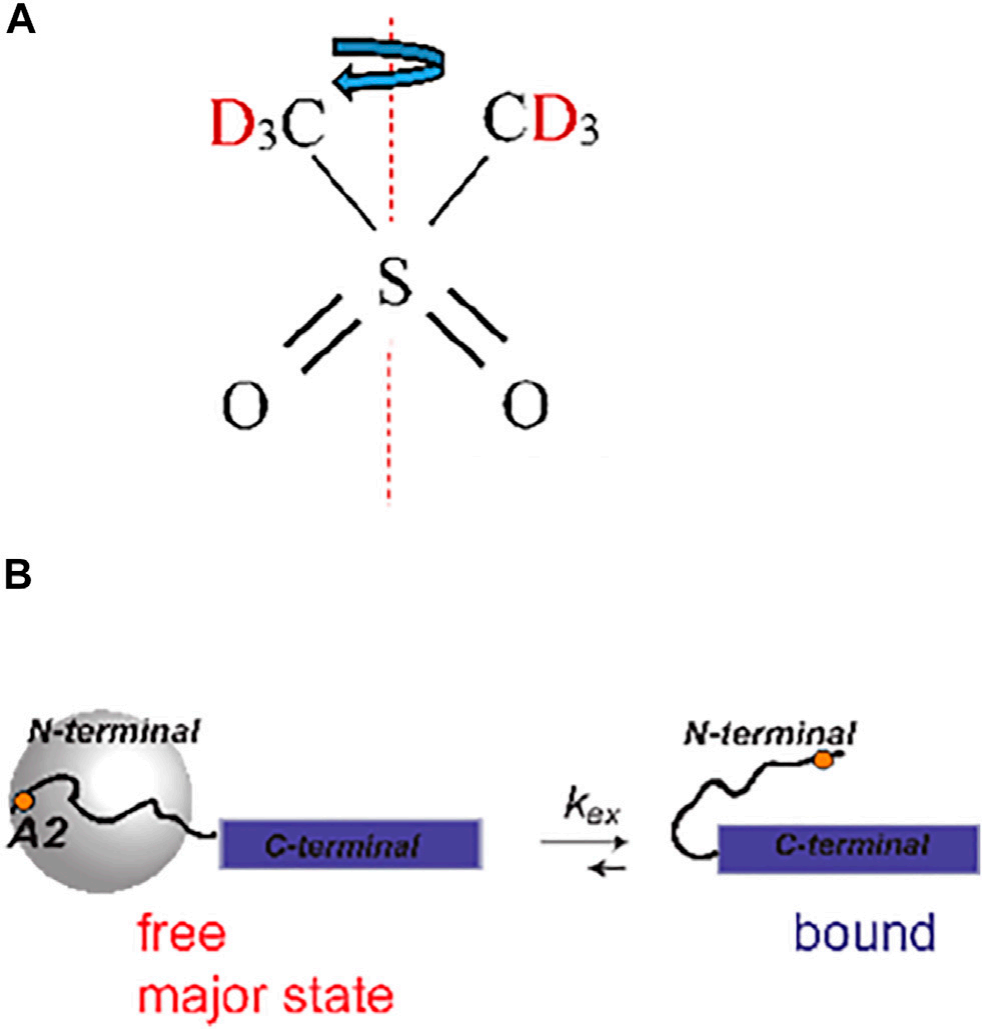
**(A)** Structure of DMS indicating the 180^°^ flipping motion around the C_2_ axis. **(B)** Slow time-scale motional modes of the A2-CD_3_ methyl group of the N-terminal domain of Aβ_1-40_ fibrils (Au et al., 2019). The disordered N-terminal domain (residues 1–16, curved line) transiently interacts with the structured C-terminal domain (blue rectangle). In the free state, the N-terminal domain undergoes large-scale diffusive motion, as represented by the gray sphere, which is absent in the bound state. The position of the A2 residue is shown as an orange dot.

Our second system is designed to test the applicability of the methods to protein samples with much lower sensitivities than small molecule compounds and with complex motional models. In particular, we employ amyloid fibril systems with monomers consisting of Aβ_1-40_ labeled at a single methyl group: the CD_3_ side chain of the A2 residue located in the beginning of the flexible N-terminal domain (spanning residues 1–16). We have previously characterized the motions of this domain at the A2-CD_3_ site using static solid-state NMR techniques (Au et al., 2019; Vugmeyster et al., 2019; Vugmeyster et al., 2020). In the hydrated state, the μs-ms motions at this site can be described by two essential processes. The main state (labeled as “free” in Figure 3B) is characterized by the pronounced large-scale fluctuations of the domain, approximated as isotropic diffusion with the diffusion coefficient *D*. They dramatically narrow the static linewidth (Figure 2) with an effective quadrupolar coupling constant of around 3 kHz. This value should be compared with the 53–55 kHz quadrupolar coupling constant expected for the methyl group without large-scale motions (Vold and Vold, 1991). There is also a minor state of the domain at around 8% of the population for the A2-CD_3_ site, in which this diffusive motion is quenched. The two states are in the conformational exchange process, with the rate constant (*k*_ex_) ranges as determined previously. The presence of the chemical exchange process was particularly evident from the dispersion pattern of ^2^H *R*_1ρ_ profiles under static conditions. (Au et al., 2019). The combined analysis of the ^2^H static rotating frame relaxation rates *R*_1ρ_, quadrupolar CPMG, and CEST calls for a more complicated model in which there is an ensemble of free states with a range of diffusion coefficients that are in conformational exchange with a single rigid bound state. Our strategy to extend the experiment to MAS conditions is to employ the simplest 2-state model of Figure 3B and assess if the fitted values of *D* and *k*_ex_ fall within the boundaries found by previous ^2^H static NMR techniques.

### Quadrupolar Chemical Exchange Saturation Transfer Experiment and RF Field Strength Calibrations

A simple quadrupolar CEST pulse sequence (Supplementary Figure S1) consists of the low-amplitude saturation pulse *ω*_RF_ (*T*, Ω) that acts on the longitudinal magnetization, followed by a non-selective 90° pulse with the same phase that brings the magnetization to the transverse plane for detection. The essence of the quadrupolar CEST is to scan the resonance offset values *Ω* corresponding to the entire spectral region(s) for all conformational states involved, which often falls into the −60–+60 kHz range for methyl groups. The saturation time *T* is chosen to optimize the efficiency of the conformational exchange and competing longitudinal relaxation. For deuteron in the methyl groups, typical values of *T* are expected to be between 1 and 40 ms. To determine the motional parameters, the RF field strength should be lower than the typical value of the quadrupolar frequencies (defined in Eq. 4 in the Theory section) in the two exchanging states. For methyl groups, the 1–5 kHz range is likely to represent the optimal conditions for most samples.

Precise calibrations of the RF field strength using the nutation experiment is complicated by two factors: the evolution of quadrupolar coupling during the nutation pulse and presence of RF inhomogeneity. The evolution of quadrupolar coupling is stronger for larger quadrupolar coupling constants and lower MAS rates (Supplementary Figure S2). The effective width of the DMS quadrupolar tensor after averaging over methyl rotations is around 55 kHz. Thus, for the 60 kHz MAS rate, the nutation experiment can be performed on the DMS central band directly with a slight correction for quadrupolar evolution. However, for the 10 and 25 kHz MAS rates, quadrupolar evolution during the nutation pulse is too pronounced and nutation can instead be performed on compounds with naturally narrower tensors. One option is to use liquid D_2_O under static conditions, utilizing the same probe as used for the compound of interest. If a solid powder sample is desirable, a good choice is hexamethyl-benzene-D_18_, whose six methyl groups participate in fast methyl jumps and 6-site jumps about the ring axis, (Vold, 1994; Gupta et al., 2015), leading to an effective quadrupolar coupling constant of about 23 kHz with an asymmetry parameter of 0.07 (Vold et al., 2009). In principle, it is also possible to perform nutation measurements directly on the fibrils sample labeled at the A2-CD_3_ site due to the narrow effective tensor in the dominant state of the fibrils at the A2 site (Figure 2B).

The RF inhomogeneity profiles can also be assessed using the nutation experiment (Figure 1A) (Gupta et al., 2015) For the 2.5 mm Bruker probe used for the 10 and 25 kHz MAS rates, powder HMB-D_18_ is the sample of choice with the sample in the rotor having a comparable length to that of DMS in the same probe. DMS is used for the 1.3 mm Bruker probe at the 60 kHz MAS rate. The RF inhomogeneity profiles (i.e., the shape of the distribution of the RF frequencies detected by the nutation experiment) are approximately proportional to the average RF frequency for a number of nominal applied RF powers. This allows us to construct a combined profile as a function of *ω*_RF_/<*ω*_RF_ > (Figure 1B), in which <*ω*_RF_ > is the weighted average over the distribution. We report <*ω*_RF_ > as the RF field strength for the CEST measurements. The inhomogeneity is rather significant and roughly comparable with the profiles reported by Gupta et al. for the 2.5 mm probe focusing on ^13^C frequency (Gupta et al., 2015). The inhomogeneity can be expected to affect the CEST measurements. Thus, the modeling procedures for the simulations of the dynamics also need to include these distributions.

### Insights From Theory and Simulations

The following matrices (plus the identity matrix) constitute a basis of the density matrix for the spin-1 system, as well as operators acting in this space: (Grey et al., 1993)

$$\begin{array}{l} {\hat{S}}_{x}=\frac{1}{2}\left(\begin{array}{ccc} 0 & 1 & 0\\ 1 & 0 & 1\\ 0 & 1 & 0 \end{array}\right),\;{\hat{S}}_{y}=\frac{1}{2}\left(\begin{array}{ccc} 0 & -i & 0\\ i & 0 & -i\\ 0 & i & 0 \end{array}\right),\;{\hat{J}}_{x}=\frac{1}{2}\left(\begin{array}{ccc} 0 & -i & 0\\ i & 0 & i\\ 0 & -i & 0 \end{array}\right),\;{\hat{J}}_{y}=\frac{1}{2}\left(\begin{array}{ccc} 0 & 1 & 0\\ 1 & 0 & -1\\ 0 & -1 & 0 \end{array}\right)\\ \;{\hat{J}}_{z}=\frac{1}{\sqrt{2}}\left(\begin{array}{ccc} 0 & 0 & -i\\ 0 & 0 & 0\\ i & 0 & 0 \end{array}\right),\hat{K}=\frac{1}{\sqrt{2}}\left(\begin{array}{ccc} 0 & 0 & 1\\ 0 & 0 & 0\\ 1 & 0 & 0 \end{array}\right),\;{\hat{S}}_{z}=\frac{1}{\sqrt{2}}\left(\begin{array}{ccc} 1 & 0 & 0\\ 0 & 0 & 0\\ 0 & 0 & -1 \end{array}\right),\hat{Q}=\frac{1}{\sqrt{6}}\left(\begin{array}{ccc} 1 & 0 & 0\\ 0 & -2 & 0\\ 0 & 0 & 1 \end{array}\right), \end{array}$$ (1)

The first row represents the single-quantum coherences, followed by the two double-quantum coherences, $\hat{K}$ and ${\hat{J}}_{z}$, $\;{\hat{S}}_{z}$ and $\hat{Q}$ stand for the Zeeman and quadrupolar order.

During the saturation period and in the frame rotating with the Larmor frequency, the secular part of the Hamiltonian is given by

$$\hat{H}=\sqrt{\frac{2}{3}}\omega_{Q}\hat{Q}+\sqrt{2}\omega_{RF}\left({\hat{S}}_{x}\cos\text{Ω}t-{\hat{S}}_{y}\sin\text{Ω}t\right)$$ (2)

where $\omega_{RF}$ is the RF field strength and Ω is its off-resonance offset. $\omega_{Q}\;$ is the frequency of the secular part of the quadrupole interaction with the angles (θ,ϕ) representing the rotation of the principal-axis system of the quadrupole interaction with respect to the laboratory frame.

$$\omega_{Q}=\frac{3\pi }{2}C_{q}\left(\frac{3{\text{cos}}^{2}\theta -1}{2}+\frac{\eta }{2}\sin^{2}\theta \cos2\phi \right)$$ (3)

The quadrupolar coupling constant is given by $C_{q}=\frac{e^{2}qQ}{h}$, and $\eta =\frac{q_{xx}-q_{yy}}{q_{zz}}\;$ represents the asymmetry of the tensor, defined in the interval 0≤η≤1 with $\left|q_{zz}\right|\geq \left|q_{yy}\right|\geq \left|q_{xx}\right|$. *eQ* is the electric quadrupole moment of the nucleus and *eq* is the largest component of the electric field gradient.

Under MAS rotation and with η=0, $\omega_{Q}\;$ becomes

$$\omega_{Q}\left(t\right)=\frac{3\pi }{4}C_{q}\left(\sqrt{2}\sin2\beta \sin\left(\omega_{MAS}t+\alpha \right)-\sin^{2}\beta \cos\left(2\omega_{MAS}t+2\alpha \right)\right)$$ (4)

where *β* and *α* are the polar and azimuthal angles with respect to the axis of rotation.

In the frame with an additional rotation with frequency Ω around the z-axis, the Hamiltonian of Eq. 2 can be transformed into the tilted frame:

$${\hat{H}}_{sec}=\sqrt{\frac{2}{3}}\omega_{Q}\hat{Q}+\sqrt{2}\omega_{RF}{\hat{S}}_{x}+\sqrt{2}\text{Ω}{\hat{S}}_{z}$$ (5)

Analogous to the off-resonance rotating frame relaxation case considered in detail for homonuclear interactions, (Rovó and Linser, 2017; Krushelnitsky et al., 2018; Rovó et al., 2019), the effect of the last two terms of Eq. 5 can be considered as an action of the effective field given by

$$\omega_{e}=\sqrt{\omega_{RF}^{2}+{\text{Ω}}^{2}}$$ (6)

For the small values of $\omega_{RF}\ll \left|\text{Ω}\right|$ employed in the CEST experiment, one expects the occurrence of rotary resonances at $\left|\text{Ω}\right|=\frac{n}{2}\omega_{MAS}$, in which *n* is an integer. The condition for the half-integer MAS rate is again analogous to homonuclear dipolar recoupling, (Rovó and Linser, 2017; Krushelnitsky et al., 2018; Rovó et al., 2019), in which it is referred to as the HORROR condition (Nielsen et al., 1994). SI1-A provides a theoretical description of the rotary resonances’ positions and relative widths based on second-order perturbation theory. The insight rendered by this theoretical description can also be demonstrated using simulations in which the Liouvillian equation (Supplementary Equation S1) is solved explicitly without any approximation. Because of the large magnitude of the quadrupolar tensor interactions with the *C*_q_ values comparable to the effective fields employed, the transformation into the tilted frame of the effective field does not lead to any simplification or render additional qualitative insights.

**FIGURE 4:**
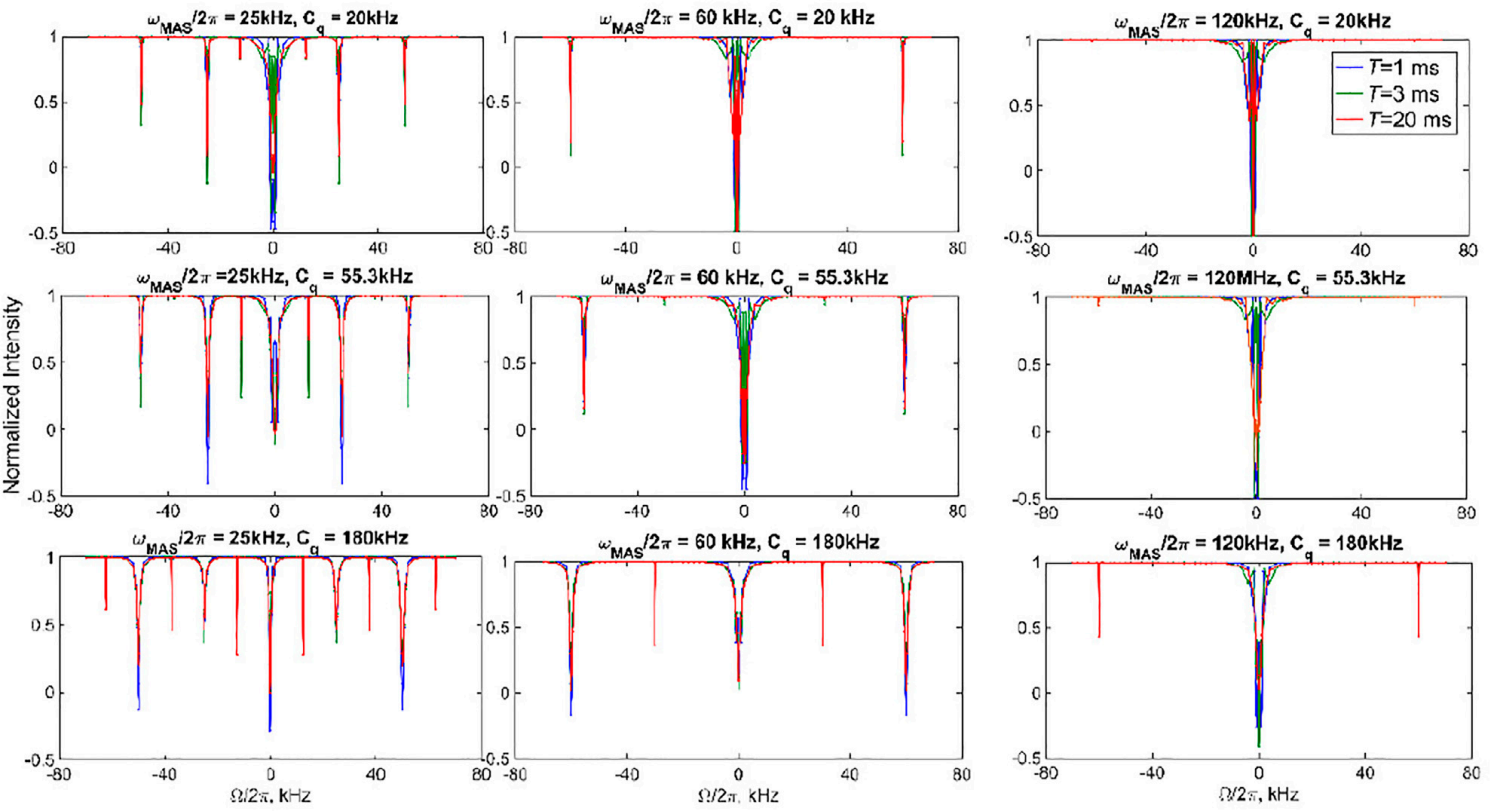
Simulated ^2^H CEST profiles in the absence of motions. The integrated intensity of all the spectral side bands normalized to intensity at *T =* 0 versus offsets Ω/2π for $\omega_{RF}/2\pi$ = 1.3 kHz and three chosen values of the saturation times *T*. Values of *C*_q_ and $\omega_{\text{MAS}}/2\pi$ are shown directly on the panels. *η* =0 in all cases.

Figure 4 demonstrates several examples of ^2^H CEST profiles corresponding to coherent contributions in the absence of motions for axially symmetric tensors with three values of *C*_q_ (20, 55, and 180 kHz), MAS rates of 25, 60, and 120 kHz, and *ω*_RF_ = 1.3 kHz. The rotary resonances are evident at the values of the offsets equal to integer and half-integer values of the MAS frequency. Their intensities are modulated by the interplay between the MAS rates and *C*_q_. The intensity of the resonances depends on the spectral intensity at Ω/2π frequencies. The half-integer resonances are much narrower (Supplementary Figure S3A) and often not as deep as the integer ones, as predicted by simple perturbation theory considerations. (Supplementary Information S1). We also explore the coherent behavior of the individual coherences of Eq. 1 for single crystallites, which demonstrates the extent of the coherent oscillations for the single and double quantum coherences and confirms the qualitative insights from perturbation theory. Supplementary Figure S3B shows an example for a single crystallite oriented at 30^o^ to the MAS axis.

As usual, motions are introduced into the Liouvillian equation by expanding the density matrix *ρ* into a direct product of the coherences (Eq. 1) and the sites corresponding to either different intra-molecular orientations of the quadrupolar tensor or changes in the value of *C*_q_ or *η* (Vold and Vold, 1991). In this direct product, the coherent evolution acts on the coherences confined to the same site, but with the site-dependent value of $\omega_{Q}$. The motions are introduced through the matrix elements between the same coherences belonging to different sites, thus encoding the model of Markovian jumps between sites. Because the rate constants of the jumps do not depend on the individual coherences, they can be represented as elements of an exchange matrix. The extended description applicable to the ^2^H CEST experiment is given in previous work (Vugmeyster et al., 2020).

**FIGURE 5:**
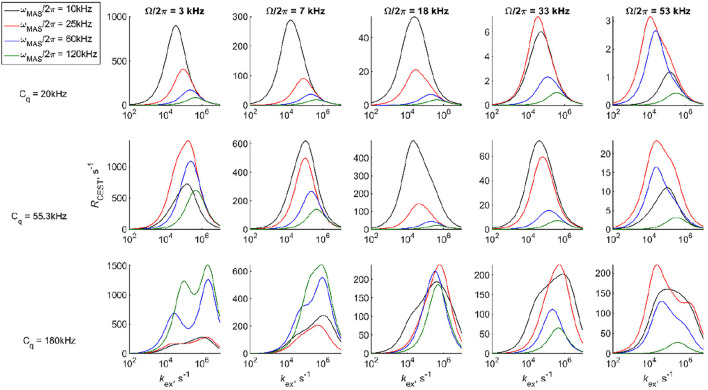
Simulated values of *R*_CEST_ versus *k*_ex_ shown for several selected offsets for the 2-site jump model with equivalent axially symmetric tensors, an equal population of sites, and a jump angle of 106°. The *R*_*CEST*_ values were obtained for integrated spectral band intensities with a single exponential approximation to the decay curves for *T* in the 0.25–128 ms range. $\omega_{RF}/2\pi$ was fixed at 1.3 kHz and the four MAS rates of 10, 25, 60, and 120 kHz were selected. The three values of *C*_q_ of 20, 55.3, and 180 kHz were chosen.

The motions induce the relaxation of the coherences. To gain insights into the effect of motions on the ^2^H CEST profiles and interplay between the values of the rate constants and MAS rate, we consider the relaxation behavior according to a simple 2-site exchange model with two axially symmetric tensors. The geometry of the motions is chosen as in Figure 3A (i.e., a jump angle of 106^°^) and three *C*_q_ values are considered (Figure 5). We select several resonance offset values, several MAS rates in the 10–120 kHz range, and saturation times *T* in 0.25–128 ms range for $\omega_{RF}/2\pi$ = 1.3 kHz. For these ranges, the magnetization decays of integrated spectral band intensities can be approximated as single exponential. The effectiveness of the relaxation, given by the relaxation rate $R_{CEST}$, is an interplay between several factors. First, the most effective relaxation is in a broad region of rate constants *k*_ex_ between 10^4^ and 10^6^ s^−1^. Second, the condition $\left|\text{Ω}\right|/2\pi <C_{q}$ is necessary for effective relaxation because it ensures significant spectral intensity at the Ω/2π frequency. Third, the $\omega_{MAS}$ dependence of the relaxation rate is heterogeneous, as it depends on the values of both $C_{q}$ and Ω. For low values of $C_{q}$, the relaxation rate decreases as $\omega_{MAS}\;$ increases, while for high $C_{q}$ values, this trend is observed only for relatively high values of Ω, but is reversed for low values of Ω. Supplementary Information S1B provides qualitative insights into the origin of these trends. Additional mechanisms affecting the ${\hat{S}}_{z}$ coherence, such as fast time-scale motions causing longitudinal relaxation, typically lead to strongly non-exponential magnetization decay curves and preclude the $R_{CEST}$ type analysis outlined here. Instead, we focus on the CEST profiles over the range of Ω values but for selected saturation times *T*.

**FIGURE 6:**
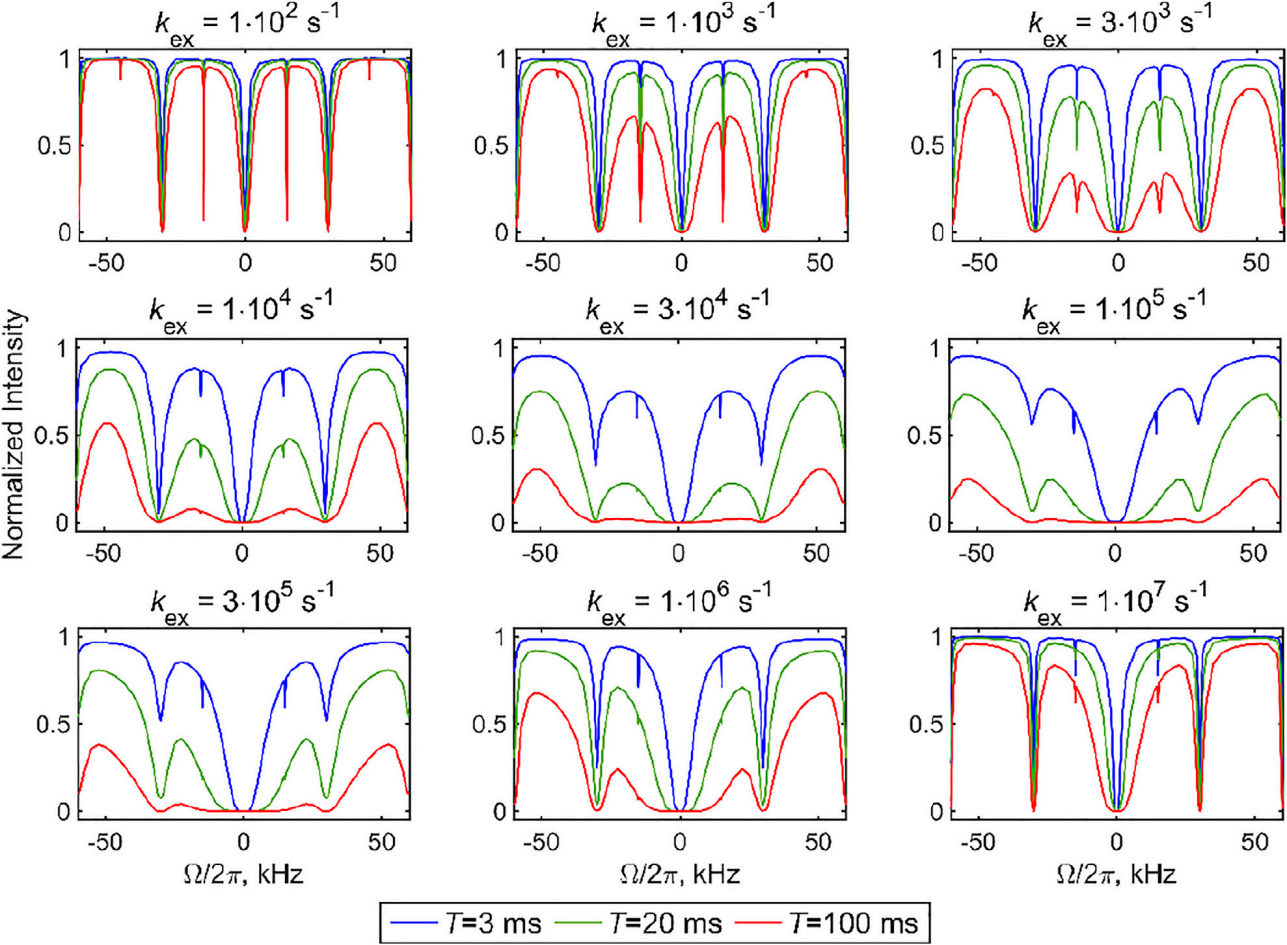
Simulated ^2^H CEST profiles for the 2-site exchange model using two axially symmetric tensors with the quadrupolar coupling constants of *C*_q_ = 55.3 kHz in both sites, equal populations, a jump angle of 106°, and values of the *k*_ex_ rate constant between 1⋅10^2^ and 1⋅10^7^ s^−1^. The integrated intensity of all the spectral side bands normalized to intensity at *T =* 0 versus offsets Ω/2π. The simulations were performed with $\omega_{\text{MAS}}/2\pi$ = 30 kHz, $\omega_{RF}/2\pi$ = 1.3 kHz, 3000 powder crystallite orientations, and three saturation times *T*. The offset sampling schedule was 2.5 kHz outside the rotary resonances and 250–500 Hz in the vicinity of the rotary resonances.

The sensitivity to *k*_ex_ can be seen directly from the simulated CEST profiles of the 2-site exchange model for two axially symmetric tensors, which display characteristic line broadening when the time scale of the exchange processes falls within the range CEST sensitivity (Figure 6). The calculations in Figure 6 are performed with the DMS tensor parameters and geometry of Figure 3A (i.e., *C*_q_ values of 55.3 kHz at both sites and a jump angle of 106°) and a fixed MAS rate of 30 kHz. The overall line broadening of the profiles falls into the 10^4^–10^6^ s^−1^ rate constant range, as expected from the analysis in Figure 5. While ample broadening is observed in the center of this range for all the resonance offset values, at the edges of the sensitivity ranges, the center region for which $\left|\text{Ω}\right|/2\pi \ll C_{q}$ is differentially broadened, especially for short saturation times. Thus, to assess the time scales of the motions, it is critical to measure different values of the saturation fields and saturation times to capture the pattern of the entire profile. Another important feature is the broadening of the coherent resonances in the presence of slow motions and consequent differential changes in intensities between the half- and full-integer rotary resonance conditions. The latter can also be useful in a qualitative assessment of whether the system falls closer to the fast or slow ends of the sensitivity range. For example, the panels corresponding to *k*_ex_ = 3⋅10^3^ s^−1^ and *k*_ex_ = 1⋅10^6^ s^−1^ are qualitatively similar in the saturation patterns, except for the first half-integer rotary resonance behavior, which is much more broadened in the *k*_ex_ = 1⋅10^6^ s^−1^ case. In general, these broadening patterns of rotary resonances are expected to be sensitive to both the values of the rate constants and the choice of the motional model, similar to the Near Rotary Resonance Relaxation Dispersion effects in rotating frame relaxation experiments (Kurauskas et al., 2017; Krushelnitsky et al., 2018; Rovó et al., 2019). Additional 2-site exchange examples are shown in Supplementary Figure S4, which includes the dependence on the MAS rate, *C*_q_ values, and unequal populations.

### Dimethyl-Sulfone Results

**FIGURE 7:**
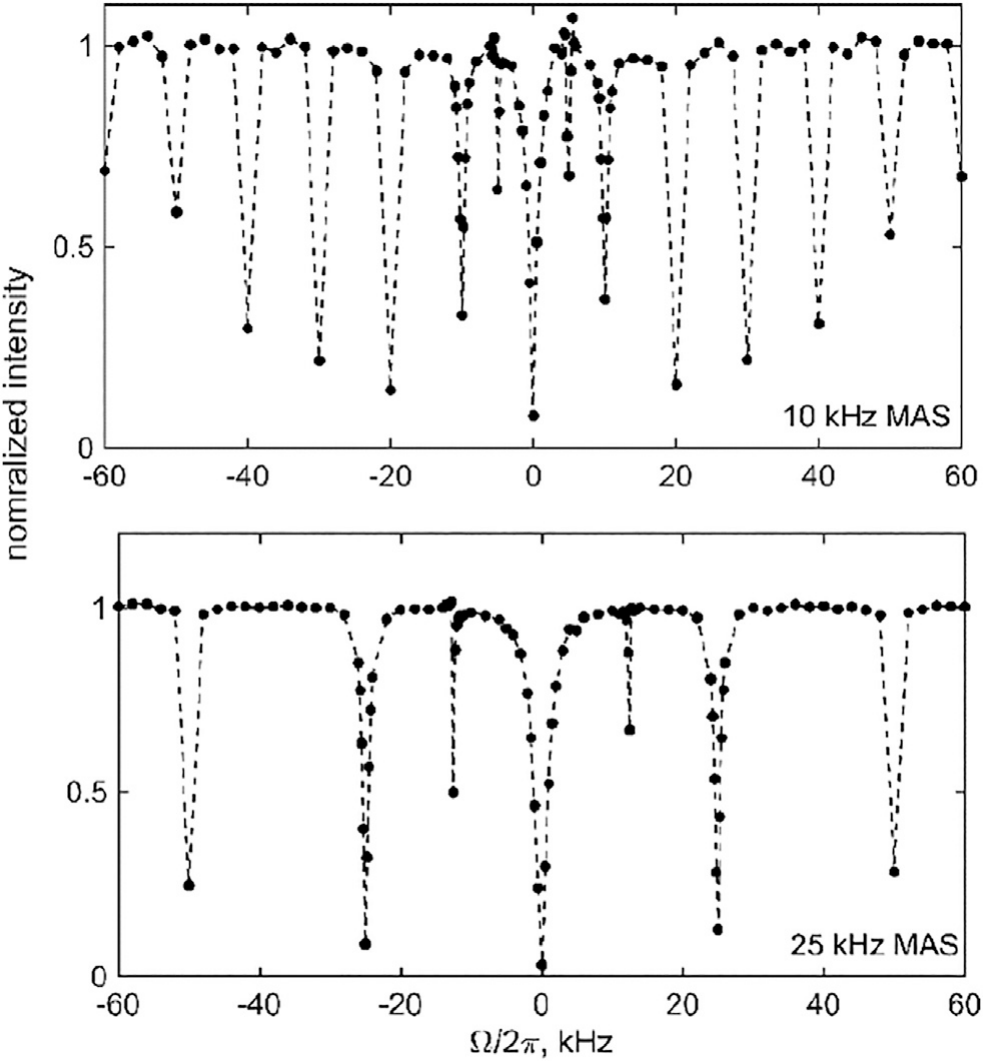
Experimental ^2^H MAS CEST profiles for DMS-D6 at 270 K and 17.6 T for the 1.3 kHz RF field strength and *T* = 3 ms. Normalized integrated central band intensity versus resonance offset Ω/2π for MAS rates of 10 kHz (upper panel) and 25 kHz (lower panel). The dotted lines are shown to guide the eye.

Before evaluating the effect of the motions on the CEST profiles of DMS-D_6_ at high temperatures at which the motions are most pronounced, we first performed the measurements at a low temperature at which the flip motion is essentially frozen. These measurements were done to confirm the effect of coherent contributions, i.e., the presence of resonances at $\pm \text{Ω }=\frac{\text{n}}{2}{\text{ω}}_{MAS}$. Figure 7 shows the ^2^H CEST profiles of DMS at 270 K with an MAS rate of either 10 or 25 kHz and using a saturation field of 1.3 kHz and a saturation time of 3 ms. The presence of integer rotary resonances is evident throughout the profiles, and the first half-integer resonance (*n* = 1) can also be seen. The width of the half-integer resonances is significantly narrower than that of the whole ones (see the theoretical considerations in SI1-A). Thus, to observe them, a dense sampling throughout the offsets is needed. We focused the dense sampling schedule on the *n* = 1 condition, i.e. ±Ω=12ωMAS, to demonstrate the principle.

The presence of multiple resonances (i.e., the coherent contributions shown in Figures 4, 7) in the CEST profiles at low MAS rates precludes the quantitative interpretation of the motional contributions of these profiles. In most cases, a compromise needs to be found between an MAS rate high enough not to render extensive resonance patterns and low enough to retain a sufficient magnitude of the unaveraged quadrupolar interaction. For DMS, we collect high temperature data at the 25 kHz MAS rate (at 76°C, 17.6 T, 2.5 mm probe) and 60 kHz MAS rate (at 55°C, 14.1 T, 1.3 mm probe) to analyze the sensitivity of the profiles to the flipping motion, which is characterized by the rate constant *k*_flip_ (Figure 3). The overall strategy is to fit the experimental data to simulations as a function of *k*_flip_ to assess whether the resulting fitted values fall within the range determined by other NMR techniques as well as evaluate the general sensitivity of the technique.

**FIGURE 8:**
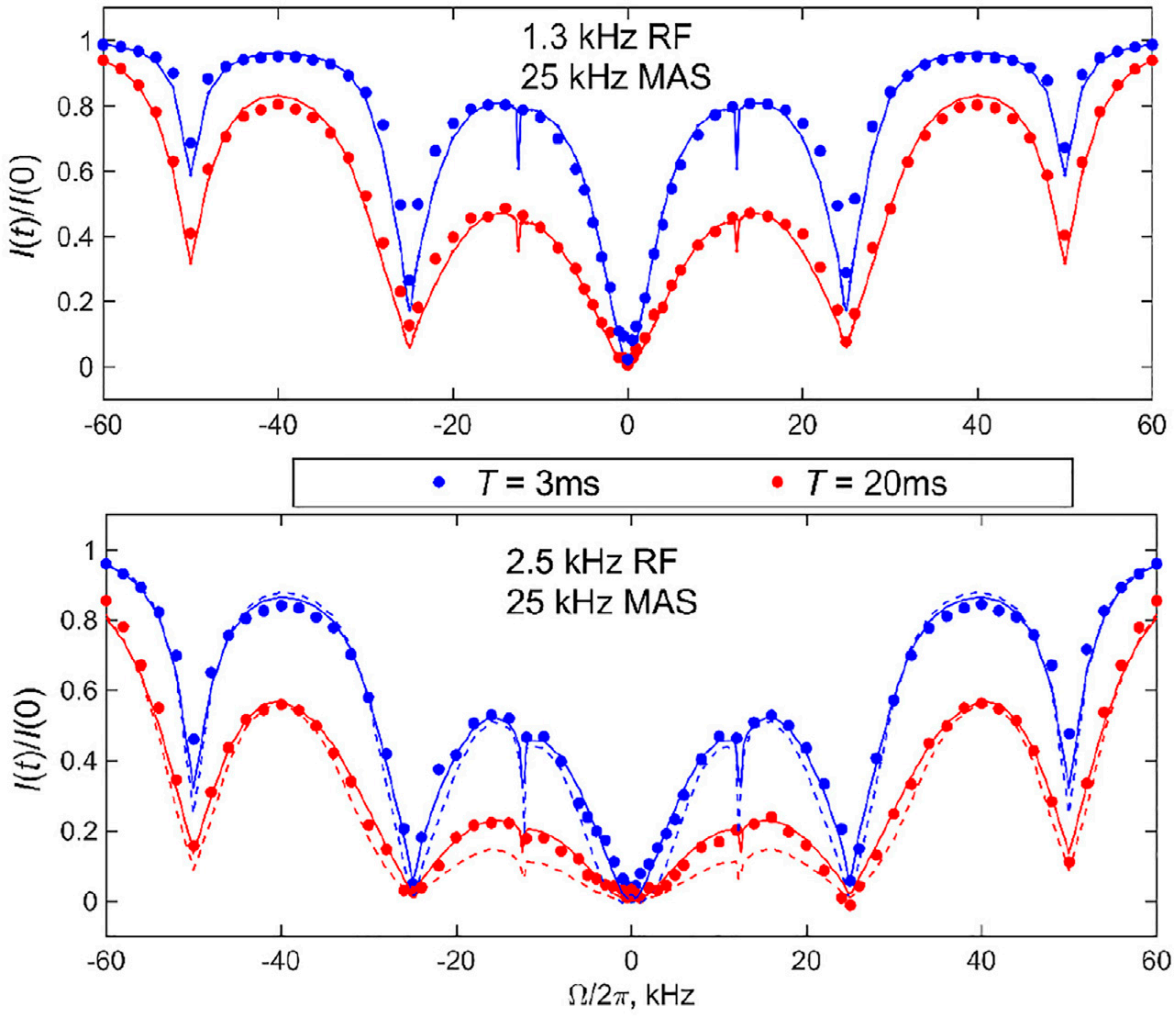
^2^H CEST center band profiles for DMS at the 25 kHz MAS rate. Experimental normalized integrated central band intensities *I*(*t*)/*I* (0) versus resonance offsets Ω/2π for saturation fields of 1.3 kHz (upper panel) and 2.5 kHz (lower panel) and saturation times of *T* =3 ms (blue circles) and *T* =20 ms (red circles) at 17.6 T and 76°C. The solid lines represent the best fit to the data, corresponding to *k*_flip_ *=* 9,000 s^−1^ for $\omega_{RF}/2\pi$ = 1.3 kHz and *k*_flip_ *=* 10,000 s^−1^ for $\omega_{RF}/2\pi$ = 2.5 kHz. The *k*_3_ value was fixed at 4.9⋅10^9^ s^−1^. The effect of RF inhomogeneity with the inhomogeneity profiles of Figure 1B was included as described in the text. The dotted lines in the bottom panel show the simulations in the absence of RF inhomogeneity.

**FIGURE 9:**
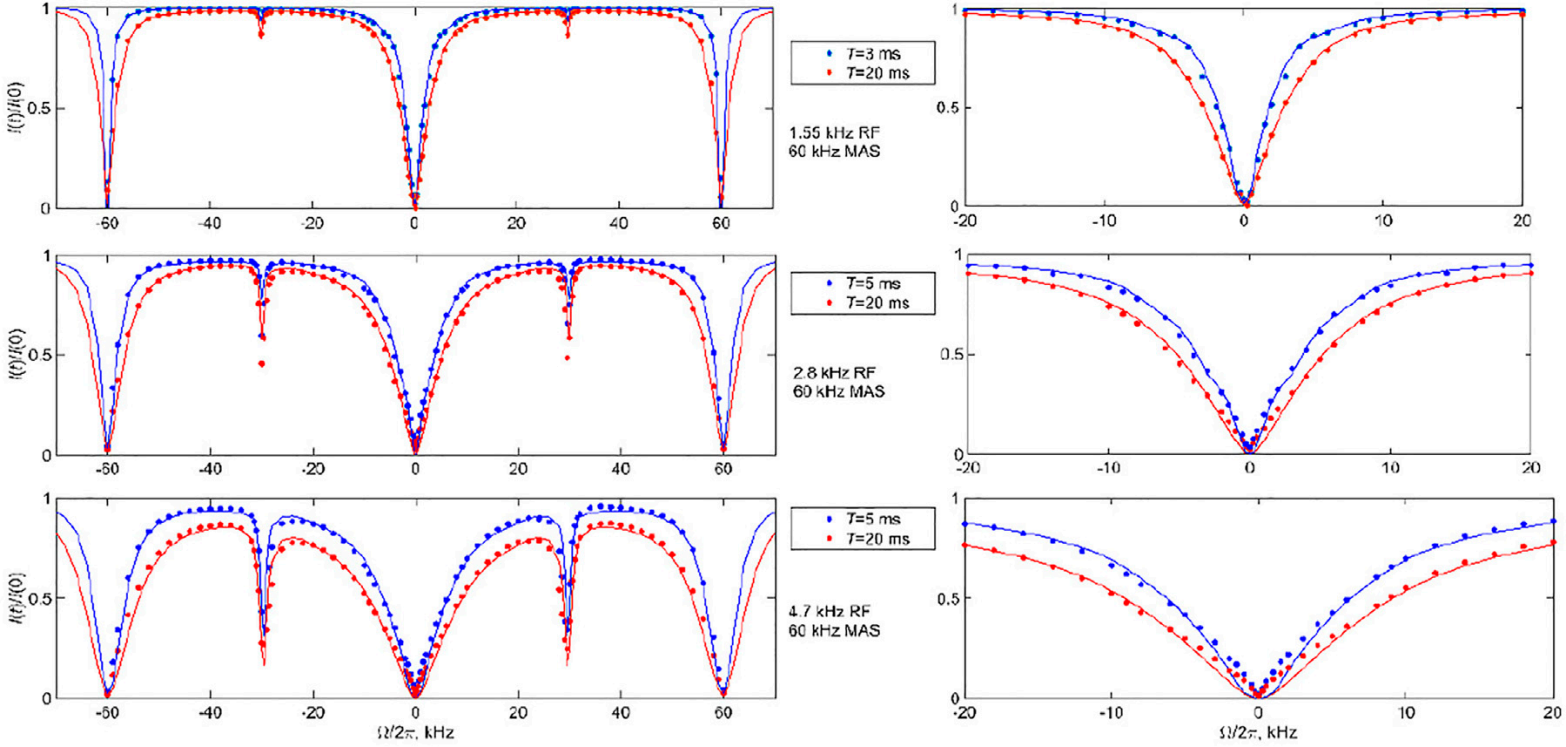
^2^H CEST central band profiles for DMS at the 60 kHz MAS rate. Experimental normalized integrated central band intensities *I*(*t*)/*I* (0) versus resonance offsets Ω/2π for the saturation fields of 1.55, 2.8, and 4.7 kHz and saturation times indicated on the panels at 14.1 T and 55°C. The right panels show the expansion of the −20–+20 kHz offset region. The solid lines represent the best fit to the data to the model of Figure 3A with a *k*_flip_ rate constant of 2,100 s^−1^. The *k*_3_ value was fixed at 3.2⋅10^9^ s^−1^. The effect of RF inhomogeneity with the inhomogeneity profiles of Figure 1B was included as described in the text.

As follows from the theoretical discussion of the 2-site jump model results (Figure 6), it is desirable to obtain the data at more than one combination of RF field strength and saturation time to cross-validate the fits and models. With high sensitivity samples such as DMS, this task is relatively easy to accomplish. We collect the data at the 25 kHz MAS rate at RF field strengths of 1.3 and 2.5 kHz and saturation times of 3 and 20 ms, with the experimental ranges of the spin-locking fields and saturation times mimicking those used to develop the CEST technique under static conditions. For the faster 60 kHz MAS rate, we utilize RF fields in the range of 1.5–4.7 kHz and saturation times between 3 and 20 ms. Figures 8, 9 present the experimental results corresponding to the central band. There is a negligible difference in the profiles when the central band results are compared with the sum of all the bands (Supplementary Figure S5).

The data were then fitted with the two-mode motional model: the slow 106^o^ flip mode between two equally populated rotamers with the corresponding rate constant *k*_flip_ (Figure 3A) and fast time-scale methyl 3-site jumps with the rate constant *k*_3_. The *k*_flip_ rate was varied and *k*_3_ value was fixed from the fits to the longitudinal relaxation times *T*_1_. The *T*_1_ times were measured with the inversion recovery pulse sequence and were 41 ms for 76°C and the 25 kHz MAS rate and 26 ms for 55°C and the 60 kHz MAS rate, corresponding to 3-site jump rate constants of 4.9⋅10^9^ s^−1^ and 3.2⋅10^9^ s^−1^, respectively. The best-fit *k*_flip_ value for the 76°C data was between 9,000 and 10,000 s^−1^, whereas it was 2,100 s^−1^ for the 55°C data (shown in Figure 8 as solid lines). These are in the range found by other techniques, particularly static ^2^H CEST (Au et al., 2019; Vugmeyster et al., 2019; Vugmeyster et al., 2020). All the simulations of the profiles included the effect of the RF inhomogeneity of the coil with the profiles of Figure 1B the CEST profiles simulated without the effect of RF inhomogeneity overestimated the saturation for offsets for which the saturation extent was significant, thus also affecting the overall shape of the profile, not only the resulting fitted rate constant. An example of one such profile simulated without inhomogeneity for $\omega_{RF}/2\pi$ = 2.5 kHz is shown by the dotted line in Figure 8.

The sensitivity of the fits to *k*_flip_ is shown in Supplementary Figures S6–S8, which allow us to assess the quality of the fits using the mean absolute difference between the experimental and simulated profiles. They demonstrate that a careful choice of RF field and saturation time delay is needed to determine the motional rate constant precisely. This is especially evident for the 60 kHz MAS data for which a wider range or RF fields are considered. At $\omega_{RF}/2\pi$ = 1.55 kHz, the 3 ms saturation time appears to be too weak to cause any significant motion dependence, while at $\omega_{RF}/2\pi$ = 2.8 and 4.7 kHz, the 5 and 20 ms saturation times both yield the desired sensitivity to the motional parameters. The effective tensor narrowing due to the fast MAS rate leads to the necessity of larger saturation times to observe the motional effects.

The data also confirm the effect of the motions on broadening the rotary resonances: the width of the full-integer rotary resonance widths is consistent between the experiment and simulations at both MAS rates. The half-integer resonances at the high temperature of 76°C at which the flipping motions are most pronounced are completely broadened for the 25 kHz MAS rate results, in accordance with the theory. The simulations show residual first half-integer rotary resonance peaks but these are too small to detect in the experiment. At the lower temperature of 55°C and high MAS rate, the first half-integer resonance is clearly visible in the data. In general, RF inhomogeneity can affect the apparent width of the rotary resonances.

**FIGURE 10:**
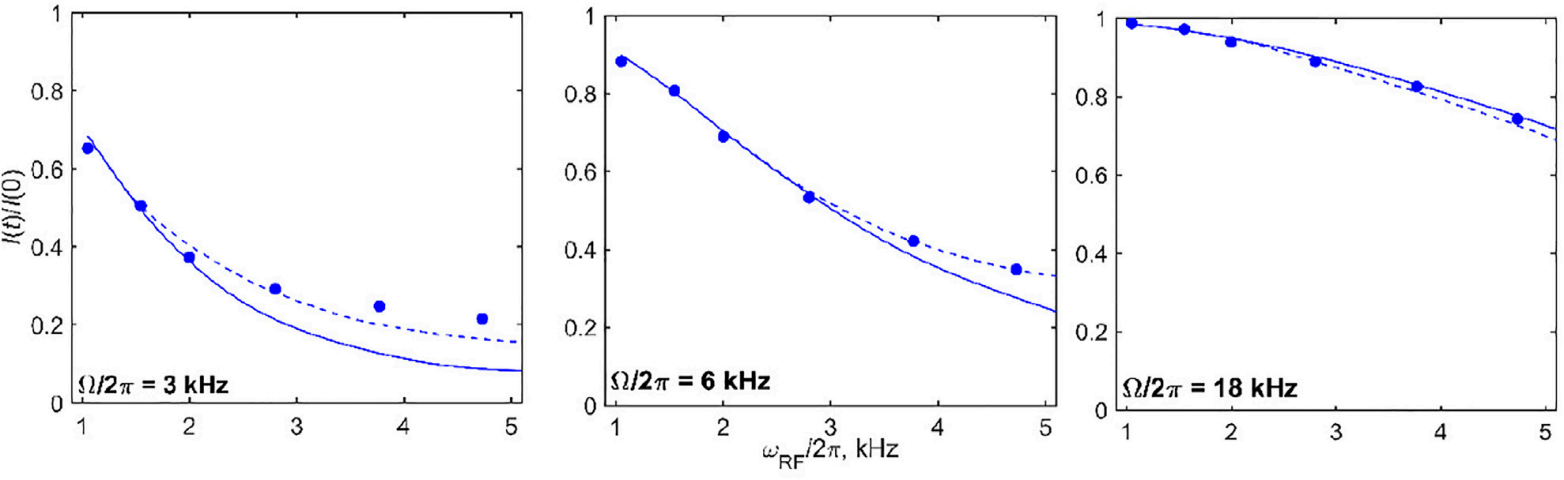
^2^H CEST DMS central band intensities *I*(*t*)/*I* (0) for selected values of offsets (shown directly on the panels) as a function of $\omega_{RF}/2\pi$ for *T*= 20 ms at the 60 kHz MAS rate and at 14.1 T and 55°C. The solid lines represent the simulations according to the model of Figure 3A with *k*_flip_ =2,100 s^−1^ and *k*_3_ = 3.2⋅10^9^ s^−1^ without the inclusion of RF inhomogeneity. The dotted lines stand for the same simulations with the inclusion of the RF inhomogeneity profiles of Figure 1B. Experimental errors are within the sizes of the symbols and were obtained as standard errors from three repeated measurements.

Additional insights can be obtained by focusing on the intensities of selected offsets for several values of $\omega_{RF}$ (Figure 10). This type of analysis can provide further confirmation of the model as well as point to the limits of validity of the approximations used to model RF inhomogeneity. In the case of DMS, at the 60 kHz MAS rate, for offset values below 3–5 kHz at which the saturation of intensities due to motions is most pronounced, the current approximation used for the simulations of inhomogeneity is likely to be somewhat imprecise. In general, however, Figure 10 demonstrates the good agreement between the modeled and experimental RF field strength dependence when the RF inhomogeneity profile of the probe is taken into account. The inhomogeneity effect is more pronounced for higher values of $\omega_{RF}$ as expected. The dynamic radial RF inhomogeneities induced by sample rotation might become relevant, (Tošner et al., 2017). They can be simulated by introducing an additional fluctuating term along ${\hat{S}}_{x}$ as a function of the phase of the MAS rotation. For the conditions of our experiment it turned out to be minor and definitely within the experimental errors. The radial inhomogeneity effects can become more important for the spin-locking of magnetization around the *x*-axis.

Overall, the analysis of the DMS profiles demonstrates that when the appropriate saturation conditions are satisfied, ^2^H CEST under MAS is a sensitive technique for the detection of slow time-scale motions. In comparison to methyl groups, for aromatic rings sites and backbone C_α_ sites, which generally correspond to $C_{q}$ = 180 kHz, the condition for optimal CEST sensitivity range may be shifted to higher MAS rates (see Figure 5), as well as toward potentially higher values of the saturation field strength. The choice of the best experimental conditions will be ultimately governed by the tensor magnitude, the time scales of motions, and tolerance of the sample toward the RF-induced heating.

### Aβ Fibril Results

**FIGURE 11:**
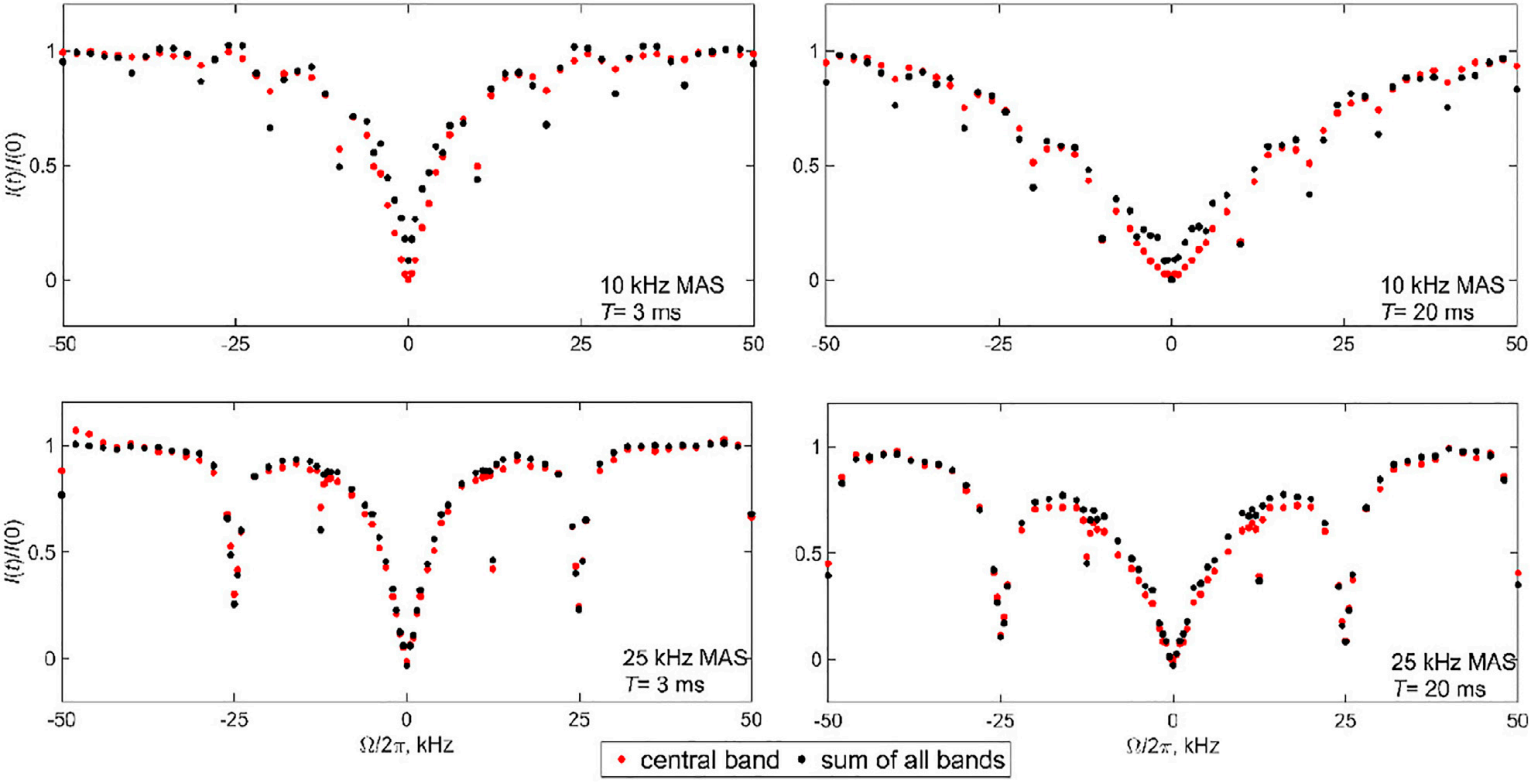
Experimental ^2^H CEST profiles of the Aβ_1-40_ fibrils labeled at the A2-CD_3_ side chain. The normalized integrated intensities *I*(*t*)/*I* (0) of the central band (red circles) are compared with the intensities of all the spectra bands shown in the spectra of Figure 2 (black circles) and are plotted as a function of the offsets Ω/2π. The saturation times and MAS rates are shown directly on the panels. The intensities *I*(*t*)/*I* (0) are plotted as a function of the offsets Ω/2π. Data were collected at 17.6 T and 37°C with $\omega_{RF}/2\pi$ = 1.3 kHz and an MAS rate of either 10 or 25 kHz.

For the Aβ fibrils labeled at the A2-CD_3_ site, due to the significant narrowing of the static linewidth in the free state of the fibrils (see the spectra in Figure 2), we choose the 10 and 25 kHz MAS conditions. The measurements were performed at a 17.6 T field strength using a 2.5 mm diameter probe and at 37°C. With significantly longer data acquisition times than for DMS, the single RF field strength of 1.3 kHz and two saturation times of 3 and 20 ms suffice (Figure 11). The overall data collection time was 5.5 days. The profiles clearly display the presence of coherent rotary resonances. The half-integer resonances are difficult to observe due to the need to implement the detailed sampling schedules necessary to catch these relatively narrow dips. We include enough offsets to observe the *n* = 1 half-integer resonances at  Ω/2π = ±12.5 kHz for the 25 kHz MAS condition to explicitly confirm their existence. The profiles are clearly sensitive to the choice of saturation time (3 or 20 ms). The *T*_1_ relaxation time of the A2 methyl group is 51 ms. For the 10 kHz MAS condition the width of the profile is somewhat dependent on whether the central band or the sum of all the bands is used. This difference is not observed for the 25 kHz MAS condition. In the discussion of the modeling and fitted parameters we will focus on the analysis for the sum of the intensities of all the side-bands and return to the potential origin of the slight differences in the profiles at the end of the section.

The modeling was performed according to the 2-state model of Figure 3B. The diffusive motion of the N-terminal domain in the free state is incorporated *via* a matrix of 192 neighboring sites on the surface of a sphere, (Au et al., 2019), with one additional site representing the bound state. To optimize the simulation time for the system with many exchanging sites, the additional mode of fast methyl 3-site jumps can be included as a phenomenological *R*_1_ term, (Vugmeyster et al., 2020), rather than introducing an additional explicit motional frame that would triple the total number of sites. Including 20 steps in each MAS period and using the model of Figure 3B are computationally demanding tasks (the details are listed in the Modeling section). The simulations took 96 h with our computational system for each MAS rate condition and a single set of the *D* and *k*_ex_ values with the inclusion of the RF inhomogeneity effect of Figure 1B.

Thus, rather than calculating a comprehensive (*D*, *k*_ex_) grid, we retained within the main range found previously using the static CEST and *R*_1ρ_ methods: the value of *D* varied from 1⋅10^6^ to 6⋅10^6^ rad^2^/s, while *k*_ex_ varied from 1⋅10^4^ to 2⋅10^5^ s^−1^. Further, instead of calculating all the profiles for the best-fit analysis, we focused on a range of characteristic offset values that can capture the widths of the pattern and resonance positions. The values were (±2, ±4, ±6, ±8, ±14, ±16, ±24, ±26 and ±10, ±20, ±30 kHz) for the 10 kHz MAS rate and (±2, ±4, ±6, ±8, ±10, ±16, ±18, ±20, and ±12.5, ±25, ±50 kHz) for the25 kHz MAS rate.

**FIGURE 12:**
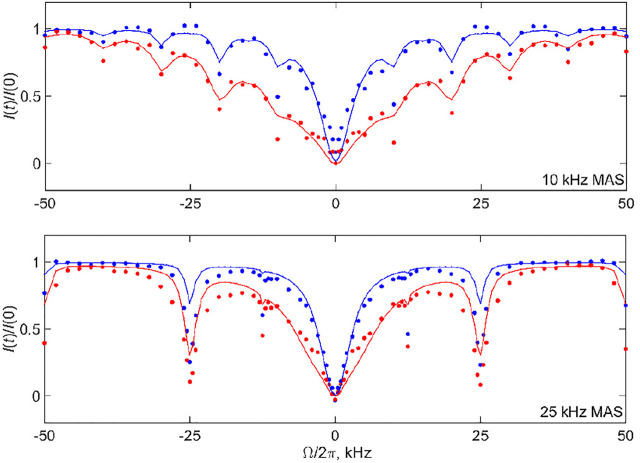
^2^H CEST profiles of the Aβ_1-40_ fibrils labeled at the A2-CD_3_ side chain corresponding to the intensities of all the bands (shown in the spectra of Figure 2) at the 10 and 25 kHz MAS rates. The experimental normalized integrated summed intensities of all the bands *I*(*t*)/*I* (0) versus resonance offsets Ω/2π for $\omega_{RF}/2\pi$ = 1.3 kHz and *T* =3 ms (blue circles) and *T* =20 ms (red circles). The lines represent the best fit to the data according to the model of Figure 3B with the following parameters: 10 kHz MAS rate (*D* = 1.7⋅10^6^ rad^2^/s, *k*_ex_ = 5⋅10^4^ s^−1^) for *T* = 3 ms and (*D* = 1.7⋅10^6^ rad^2^/s, *k*_ex_ = 1⋅10^5^ s^−1^) for *T* = 20 ms; 25 kHz MAS rate (*D* = 1.0⋅10^6^ rad^2^/s, *k*_ex_ = 2⋅10^4^ s^−1^) for *T* = 3 ms and (*D* = 1.7⋅10^6^ rad^2^/s, *k*_ex_ = 3⋅10^4^ s^−1^) for *T* = 20 ms. Data were collected at 17.6 T and 37°C. The effect of RF inhomogeneity with the inhomogeneity profiles of Figure 1B was included as described in the text.

The mean absolute differences of these searches are shown in Supplementary Figure S9. There are shallow minima around the best-fit parameters as follows: for the 10 kHz MAS rate, the values are *D*= 1.7⋅10^6^ rad^2^/s and *k*_ex_ = 5⋅10^4^ s^−1^ for *T* = 3 ms and *D* = 1.7⋅10^6^ rad^2^/s and *k*_ex_ = 1⋅10^5^ s^−1^ for *T* = 20 ms, while for the 25 kHz MAS rate, the values are *D* = 1.0⋅10^6^ rad^2^/s and *k*_ex_ = 2⋅10^4^ s^−1^ for *T* = 3 ms and *D* = 1.7⋅10^6^ rad^2^/s and *k*_ex_ = 3⋅10^4^ s^−1^ for *T* = 20 ms. These fits are demonstrated in Figure 12 by the solid lines. There is a positive correlation between the *D* and *k*_ex_ values, which can be rationalized by the fact that fast diffusion narrows the overall CEST pattern, whereas relatively slow conformational exchange widens it. This correlation, which was also noted and analyzed in more detail for the static case, (Vugmeyster et al., 2020), leads to the whole subset of relatively comparable (*D*, *k*_ex_) pairs in terms of the quality of the fits. The shallow minima chosen for the profiles in Figure 12 are the result of the compromise between matching the overall width of the pattern across all the offsets and the intensities at the coherent resonances. We have performed fits for individual data sets rather than the combined fit in order to determine the ranges of acceptable parameters within the limitation of the model and correlations between the fitted value of *D* and *k*_ex_. If the global fit is performed (Supplementary Material S10), the best-fit parameters are *D =* 1.7⋅10^6^ rad^2^/s, *k*_ex_ = 3⋅10^4^ s^−1^.

The presence of rotary resonances in the data is not trivial, as it confirms from another angle the existence of the slow conformational exchange between the free and bound states of the fibrils. As demonstrated in Supplementray Figure S11, the diffusion mode alone can match the width of the narrow central region in the −6–+6 kHz range. However, it not only misses the overall outer width and shape of the pattern [which was also observed for the static data, see Supplementray Figure S5 of prior work (Vugmeyster et al., 2020)], but also broadens any traces of the coherent resonances. Supplementray Figure S12 demonstrates the alternative fits for the 10 kHz MAS rate, *T*= 20 ms profile using three (*D*, *k*_ex_) pairs, including the best-fit profile of Figure 12. The parameters (*D =* 6.0⋅10^6^ rad^2^/s, *k*_ex_ = 2⋅10^5^ s^−1^) lead to a comparable quality of the overall fit judging by the grid search. However, the dips in intensities at the resonance positions are underestimated compared with the best fit results of Figure 12. By contrast, the parameters (*D =* 1.0⋅10^6^ rad^2^/s, *k*_ex_ = 5⋅10^4^ s^−1^) capture the intensities at the resonances somewhat better at the expense of matching the overall width of the pattern. Thus, the optimal values of *k*_ex_ for observing the existence of coherent resonances fall into the 3–5⋅10^4^ s^−1^ range. For the 25 kHz MAS rate and *T*= 20 ms profile, Supplementray Figure S12B shows how the intensity at the first resonance varies for different pairs of (*D*, *k*_ex_). While none of them capture the experimental intensity directly, the closest match is seen for *k*_ex_ in the 2–5⋅10^4^ s^−1^ range, while there is relatively weak dependence on the value of *D*. As half-integer rotary resonances could be difficult to observe, for most cases of low sensitivity samples the observation of the first full-integer resonance’s intensity and width will be sufficient for constraining the model and its parameters.

Our fits clarify that the model is far from perfect in catching the exact intensities at the resonances. The first half-integer resonances evident in the experimental data are completely absent for both MAS conditions and the first integer resonance is underestimated. This implies that the exchange processes in the fibrils are likely to be more complex than in the simplified model of Figure 3B. Nonetheless, within the precision of the analysis, we confirm the qualitative presence of the conformational exchange and its time scale. Another hint that our model is an oversimplification is suggested by the slight discrepancy between the CEST profiles of the central band and profiles for the sum of all the bands in the 10 kHz MAS data of Figure 6. These discrepancies may reflect the existence of more complex ensembles of free and bound states of the N-terminal subdomain of the fibrils. A previous combined analysis of static ^2^H NMR CEST, rotating frame *R*_1ρ_ rates, and QCPMG data of the N-terminal subdomain indicated a complex conformational space, corresponding to an ensemble of conformations for the free state in exchange with a single bound state (Au et al., 2019; Vugmeyster et al., 2019; Vugmeyster et al., 2020). The ensemble is characterized by clusters of *D* values around 1–3⋅10^6^ rad^2^/s, and 1⋅10^8^ rad^2^/s, with corresponding *k*_ex_ values clustered in 0.1–1⋅10^5^ and 1–3⋅10^6^ s^−1^. The values from the new MAS measurements are in line with these previously determined ranges.

The overall strategies presented for DMS and Aβ fibrils will hold for a variety of biological systems including protein aggregates, complexes, and crystals. Simpler NMR measurements such as line shape analysis can serve as a complementary tool to narrow some of the expected time scale ranges. The determination of the models for this complex systems should start with the simplest scenarios of limited number of exchanging sites, and increase in the level of complexity when governed by the experimental data. Future improvements in the speed of computations are expected to greatly benefit model selection procedures.

## Conclusion

The analyses of the results of DMS-D_6_ with a simple 2-site rotameric flip model with known parameters as well as the Aβ_1−40_ fibril sample with a complex model previously assessed by other techniques indicated that the ^2^H CEST experiment can quantify the slow motional modes in rotating solids. For the best precision and motional model development, it is desirable to perform the measurements for more than one combination of the saturation fields and saturation times. Special attention should be paid to the examination of the experimental and simulated intensities at rotary resonance positions, as they can pinpoint to details of motional regimes and mechanisms. This is an additional strength of rotating versus static approach. Assessing probe RF inhomogeneity can be important for improving the accuracy of the results. For low sensitivity protein samples with complex models, MAS rates, saturation field strength, and saturation times must be selected carefully to optimize data collection strategies. Further, the explicit modeling procedures for complex models have to be computationally optimized to render them friendly for model and parameter selection. Once these strategies are in place, the ^2^H CEST technique can be a powerful tool for studies of protein dynamics.

## Data Availability

The raw data supporting the conclusions of this article will be made available by the authors, without undue reservation.

## References

[B1] Akbey Ü. Nieuwkoop A. J. Wegner S. Voreck A. Kunert B. Bandara P. (2014). Quadruple-resonance Magic-Angle Spinning NMR Spectroscopy of Deuterated Solid Proteins. Angew. Chem. Int. Ed. 53, 2438–2442. 10.1002/anie.201308927 24474388

[B2] Al-Mohy A. H. Higham N. J. (2010). A New Scaling and Squaring Algorithm for the Matrix Exponential. SIAM J. Matrix Anal. Appl. 31, 970–989. 10.1137/09074721x

[B3] Au D. F. Ostrovsky D. Fu R. Vugmeyster L. (2019). Solid-state NMR Reveals a Comprehensive View of the Dynamics of the Flexible, Disordered N-Terminal Domain of Amyloid-β Fibrils. J. Biol. Chem. 294, 5840–5853. 10.1074/jbc.ra118.006559 30737281 PMC6463690

[B4] Bain A. D. Berno B. (2011). Liouvillians in NMR: the Direct Method Revisited. Prog. Nucl. Magn. Reson. Spectrosc. 59, 223–244. 10.1016/j.pnmrs.2010.12.002 21920219

[B5] Bjerring M. Paaske B. Oschkinat H. Akbey Ü. Nielsen N. C. (2012). Rapid Solid-State NMR of Deuterated Proteins by Interleaved Cross-Polarization from 1H and 2H Nuclei. J. Magn. Reson. 214, 324–328. 10.1016/j.jmr.2011.10.020 22130517

[B6] Bouvignies G. Kay L. E. (2012). Measurement of Proton Chemical Shifts in Invisible States of Slowly Exchanging Protein Systems by Chemical Exchange Saturation Transfer. J. Phys. Chem. B 116, 14311–14317. 10.1021/jp311109u 23194058

[B7] Brown M. J. Void R. L. Hoatson G. L. (1996). Selective Inversion Investigations of Slow Molecular Motion in Solid State Deuteron NMR Spectroscopy. Solid State. Nucl. Magn. Reson. 6, 167–185. 10.1016/0926-2040(95)01213-3 8784956

[B8] Favre D. E. Schaefer D. J. Chmelka B. F. (1998). Direct Determination of Motional Correlation Times by 1D MAS and 2D Exchange NMR Techniques. J. Magn. Reson. 134, 261–279. 10.1006/jmre.1998.1506 9761702

[B9] Frydman L. Vallabhaneni S. Lee Y. K. Emsley L. (1994). Solid‐State Dynamic Processes in Complex Systems Analyzed by Two‐dimensional Isotropic-Anisotropic Correlation Nuclear Magnetic Resonance. J. Chem. Phys. 101, 111–117. 10.1063/1.468185

[B10] Gérardy-Montouillout V. Malveau C. Tekely P. Olender Z. Luz Z. (1996). ODESSA, a New 1D NMR Exchange experiment for Chemically Equivalent Nuclei in Rotating Solids. J. Magn. Reson. Ser. A 123, 7–15. 10.1006/jmra.1996.0208 8980058

[B11] Grey C. P. Veeman W. S. W. Vega A. J. A. (1993). Rotational echo14N/13C/1H Triple Resonance Solid‐state Nuclear Magnetic Resonance: A Probe of13C-14N Internuclear Distances. J. Chem. Phys. 98, 7711–7724. 10.1063/1.464579

[B12] Gupta R. Hou G. Polenova T. Vega A. J. (2015). RF Inhomogeneity and How it Controls CPMAS. Solid State. Nucl. Magn. Reson. 72, 17–26. 10.1016/j.ssnmr.2015.09.005 26422256 PMC4674349

[B13] Higham N. J. (2005). The Scaling and Squaring Method for the Matrix Exponential Revisited. SIAM J. Matrix Anal. Appl. 26, 1179–1193. 10.1137/04061101x

[B14] Jain S. K. Nielsen A. B. Hiller M. Handel L. Ernst M. Oschkinat H. (2014). Low-power Polarization Transfer Between Deuterons and Spin-1/2 Nuclei Using Adiabatic RESPIRATIONCP in Solid-State NMR. Phys. Chem. Chem. Phys. 16, 2827–2830. 10.1039/c3cp54419b 24418905

[B15] Krushelnitsky A. Zinkevich T. Reif B. Saalwächter K. (2014). Slow Motions in Microcrystalline Proteins as Observed by MAS-Dependent 15N Rotating-Frame NMR Relaxation. J. Magn. Reson. 248, 8–12. 10.1016/j.jmr.2014.09.007 25282442

[B16] Krushelnitsky A. Gauto D. Rodriguez Camargo D. C. Schanda P. Saalwächter K. (2018). Microsecond Motions Probed by Near-Rotary-Resonance R1ρ 15N MAS NMR Experiments: the Model Case of Protein Overall-Rocking in Crystals. J. Biomol. NMR 71, 53–67. 10.1007/s10858-018-0191-4 29845494 PMC5986846

[B17] Kurauskas V. Izmailov S. A. Rogacheva O. N. Hessel A. Ayala I. Woodhouse J. (2017). Slow Conformational Exchange and Overall Rocking Motion in Ubiquitin Protein Crystals. Nat. Commun. 8, 145. 10.1038/s41467-017-00165-8 28747759 PMC5529581

[B18] Matlahov I. Van der Wel P. C. A. (2018). Hidden Motions and Motion-Induced Invisibility: Dynamics-Based Spectral Editing in Solid-State NMR. Methods 148, 123–135. 10.1016/j.ymeth.2018.04.015 29702226 PMC6133742

[B19] Nielsen N. C. Bildso/E H. Jakobsen H. J. Levitt M. H. (1994). Double‐quantum Homonuclear Rotary Resonance: Efficient Dipolar Recovery in Magic‐Angle Spinning Nuclear Magnetic Resonance. J. Chem. Phys. 101, 1805–1812. 10.1063/1.467759

[B20] Palmer A. G. Koss H. (2019). “Chemical Exchange,” in Methods in Enzymology. Editor Wand A. J. (Academic Press), 177–236. 10.1016/bs.mie.2018.09.028 PMC7493007 30638530

[B21] Palmer A. G. 3rd (2014). Chemical Exchange in Biomacromolecules: Past, Present, and Future. J. Magn. Reson. 241, 3–17. 10.1016/j.jmr.2014.01.008 24656076 PMC4049312

[B22] Quinn C. M. McDermott A. E. (2012). Quantifying Conformational Dynamics Using Solid-State R1ρ Experiments. J. Magn. Reson. 222, 1–7. 10.1016/j.jmr.2012.05.014 22820004 PMC3572234

[B23] Rovó P. (2020). Recent Advances in Solid-State Relaxation Dispersion Techniques. Solid State. Nucl. Magn. Reson. 108, 101665. 10.1016/j.ssnmr.2020.101665 32574905

[B24] Rovó P. Linser R. (2017). Microsecond Time Scale Proton Rotating-Frame Relaxation under Magic Angle Spinning. J. Phys. Chem. B 121, 6117–6130. 10.1021/acs.jpcb.7b03333 28534618

[B25] Rovó P. Linser R. (2018). Microsecond Timescale Protein Dynamics: a Combined Solid-State NMR Approach. ChemPhysChem 19, 34–39. 10.1002/cphc.201701238 29149466

[B26] Rovó P. Smith C. A. Gauto D. De Groot B. L. Schanda P. Linser R. (2019). Mechanistic Insights into Microsecond Time-Scale Motion of Solid Proteins Using Complementary 15N and 1H Relaxation Dispersion Techniques. J. Am. Chem. Soc. 141, 858–869. 10.1021/jacs.8b09258 30620186 PMC6982537

[B27] Saalwächter K. Fischbach I. (2002). The Application of MAS Recoupling Methods in the Intermediate Motional Regime. J. Magn. Reson. 157, 17–30. 10.1006/jmre.2002.2552 12202129

[B28] Siemer A. B. Huang K.-Y. Mcdermott A. E. (2010). Protein-Ice Interaction of an Antifreeze Protein Observed With Solid-State NMR. Proc. Natl. Acad. Sci. USA 107, 17580–17585. 10.1073/pnas.1009369107 20884853 PMC2955146

[B29] Tošner Z. Purea A. Struppe J. O. Wegner S. Engelke F. Glaser S. J. (2017). Radiofrequency Fields in MAS Solid State NMR Probes. J. Magn. Reson. 284, 20–32. 10.1016/j.jmr.2017.09.002 28946058

[B30] Vallurupalli P. Bouvignies G. Kay L. E. (2012). Studying "invisible" Excited Protein States in Slow Exchange With a Major State Conformation. J. Am. Chem. Soc. 134, 8148–8161. 10.1021/ja3001419 22554188

[B31] Van der Wel P. C. A. (2017). Insights into Protein Misfolding and Aggregation Enabled by Solid-State NMR Spectroscopy. Solid State. Nucl. Magn. Reson. 88, 1–14. 10.1016/j.ssnmr.2017.10.001 29035839 PMC5705391

[B32] Vold R. R. Vold R. L. (1991). “Deuterium Relaxation in Molecular Solids,” in Advances in Magnetic and Optical Resonance. Editor Warren W. (San Diego: Acadenic Press), 85–171. 10.1016/b978-0-12-025516-0.50006-1

[B33] Vold R. L. Hoatson G. L. Vugmeyster L. Ostrovsky D. De Castro P. J. (2009). Solid State Deuteron Relaxation Time Anisotropy Measured with Multiple echo Acquisition. Phys. Chem. Chem. Phys. 11, 7008–7012. 10.1039/b907343d 19652835

[B34] Vold R. R. (1994). “Deuterium NMR Studies of Dynamics in Solids and Liquid Crystals,” in Nuclear Magnetic Resonance Probes of Molecular Dynamics. Editor Tycko R. (Dordrecht: Kluwer academic Publishers), 27–112. 10.1007/978-94-011-1410-3_2

[B35] Vugmeyster L. Ostrovsky D. (2019). Deuterium Rotating Frame NMR Relaxation Measurements in the Solid State under Static Conditions for Quantification of Dynamics. Chemphyschem 20, 333–342. 10.1002/cphc.201800454 30079456 PMC6499496

[B36] Vugmeyster L. Au D. F. Ostrovsky D. Fu R. (2019). Deuteron Solid‐State NMR Relaxation Measurements Reveal Two Distinct Conformational Exchange Processes in the Disordered N‐Terminal Domain of Amyloid‐β Fibrils. ChemPhysChem 20, 1680–1689. 10.1002/cphc.201900363 31087613 PMC6663588

[B37] Vugmeyster L. Ostrovsky D. Fu R. (2020). Deuteron Quadrupolar Chemical Exchange Saturation Transfer (Q‐CEST) Solid‐State NMR for Static Powder Samples: Approach and Applications to Amyloid‐β Fibrils. ChemPhysChem 21, 220–231. 10.1002/cphc.201901053 31805217 PMC7002291

