# Supplementary material for: Deuteron Chemical Exchange Saturation Transfer for the Detection of Slow Motions in Rotating Solids
Source: Front Mol Biosci. 2021 Jul 27;8:705572. doi: 10.3389/fmolb.2021.705572 (PMC8353179; doi:10.3389/fmolb.2021.705572)
Supplement: Supplementary file 1 [file DataSheet1.PDF]

Supplementary Material: Deuteron Chemical Exchange Saturation Transfer for the Detection of Slow Motions in Rotating Solids

Liliya Vugmeyster,[a,*] Dmitry Ostrovsky,[b] Alexander Greenwood,[c] Riqiang Fu[d]

[a] *Department of Chemistry, University of Colorado Denver, Denver CO USA 80204*

[b] *Department of Mathematics, University of Colorado Denver, Denver CO USA 80204*

[c] *Department of Chemistry,University of Cincinnati, Cincinnati, OH 45221*

[d] *National High Field Magnetic Laboratory, Tallahassee, FL USA 32310*

[a,*] Department of Chemistry, University of Colorado at Denver, 1201 Larimer St, Denver, CO 80204; email: LILIYA.VUGMEYSTER@UCDENVER.EDU

SI1

A). Theoretical description of the rotary resonances

The Liouville–von Neumann equation(Bain and Berno, 2011) is

$\frac{d\rho}{dt} = L\rho$ (no double hats above the symbols anymore) (Eq. S1)

$L = L_0 + \sqrt{2}\omega_{RF}S_x$, where L_0 includes the terms stemming from the $\hat{Q}$ and $\hat{S}_z$ interactions and S_x is a superoperator.

In the following discussion, we consider $\omega_{RF} \ll |\Omega|, \omega_{MAS}$ as applicable to the ^{2}H CEST experiment.

The eigenvectors and eigenvalues of L_0 are $(\hat{S}_z, 0)$, $(\hat{Q}, 0)$, $(\hat{S}_\pm^{(+)} = \frac{i}{2}(\hat{S}_x \pm i\hat{S}_y \pm i\hat{J}_x + \hat{J}_y), \pm i(\omega_Q + \Omega))$, $(\hat{S}_\pm^{(-)} = \frac{i}{2}(\hat{S}_x \mp i\hat{S}_y \pm i\hat{J}_x - \hat{J}_y), \pm i(\omega_Q - \Omega))$, and $(\hat{K}_\pm = \frac{1}{\sqrt{2}}(\hat{K} \pm i\hat{J}_z), \pm i2\Omega)$. On the basis of the eigenvectors of L_0, $\rho_k = (\hat{S}_\pm^{(+)}, \hat{S}_\pm^{(-)}, \hat{K}_\pm, \hat{S}_z, \hat{Q})$. We refer to the corresponding eigenvalues λ_k, $\lambda_k = i(\omega_Q + \Omega, -\omega_Q - \Omega, \omega_Q - \Omega, -\omega_Q + \Omega, 2\Omega, -2\Omega, 0, 0)$. For the MAS conditions, several λ_k values become time-dependent, but the eigenvectors remain the same as in the static case, as they do not depend on ω_Q. The eigenvalues can be represented as $\lambda_k = i(q_k\omega_Q + z_k\Omega)$, where $q_k = -1,0,1$ and $z_k = -2,-1,0,1,2$ as appropriate.

In this basis, the superoperator $\sqrt{2}S_x$ is

$$\delta L = \begin{pmatrix} 0 & 0 & 0 & 0 & -1/\sqrt{2} & 0 & -1/2 & -\sqrt{3}/2 \\ 0 & 0 & 0 & 0 & 0 & -1/\sqrt{2} & -1/2 & -\sqrt{3}/2 \\ 0 & 0 & 0 & 0 & 0 & -1/\sqrt{2} & 1/2 & -\sqrt{3}/2 \\ 0 & 0 & 0 & 0 & 1/\sqrt{2} & 0 & -1/2 & \sqrt{3}/2 \\ 1/\sqrt{2} & 0 & 0 & -1/\sqrt{2} & 0 & 0 & 0 & 0 \\ 0 & 1/\sqrt{2} & 1/\sqrt{2} & 0 & 0 & 0 & 0 & 0 \\ 1/2 & 1/2 & -1/2 & 1/2 & 0 & 0 & 0 & 0 \\ \sqrt{3}/2 & \sqrt{3}/2 & \sqrt{3}/2 & -\sqrt{3}/2 & 0 & 0 & 0 & 0 \end{pmatrix} \quad \text{(Eq. S2)}$$

We can expand the solution of Eq. (S1) in the basis of ρ_k with the evolution due to L_0 explicitly written:

$$\rho(t) = \sum_k a_k(t) e^{i\Phi_k(t)} \rho_k$$

where $\Phi_k(t) = \int \lambda_k(t)dt = q_k\Phi_0(t) + z_k\Omega t$, with

$$\Phi_0(t) = \int \omega_Q(t)dt = \frac{3\pi}{4}\frac{C_q}{\omega_{MAS}}\left(-\sqrt{2}\sin 2\beta\cos(\omega_{MAS}t + \alpha) - \frac{1}{2}\sin^2\beta\sin(2\omega_{MAS}t + 2\alpha)\right)$$

The time-dependent coefficients $a_k(t)$ satisfy

$$a_l' = \omega_{RF}\sum_k \delta L_{lk}\, e^{i\Phi_k(t) - i\Phi_l(t)} a_k(t)$$

We consider the coefficients $a_k(t)$, which are slowly changing on the time scale of the single MAS rotation (in analogy to the average Hamiltonian theory)(Haeberlen and Waugh, 1968;Brinkmann, 2016) and expand the coefficients by the orders of ω_{RF}.

$a_k^{(0)} = \delta_{k,7}$ (this is the initial condition, only the $\hat{S}_z$ coherence is present.)

$$a_k^{(1)}(t) = \omega_{RF}\,\delta L_{k,7}\int_0^t e^{-i\Phi_k(t_1)}dt_1 \quad \text{(Eq. S3)}$$

where $\Phi_7(t) = 0$ is taken to account. In the first order in ω_{RF}, the only possible transitions are between $\hat{S}_z$ and the single-quantum coherences with *k* = 1,..., 4, for which $q_k = \pm 1, z_k = \pm 1$. The main insight is that of the two components of $\Phi_k(t)$, one, $\Phi_0(t)$, is periodic with the time of the single MAS rotation period and another, Ωt, is not. Thus, every rotation produces an additional $e^{2\pi i\frac{\Omega}{\omega_{MAS}}}$ multiplicative factor in the integral. For the perturbative terms $\sim\omega_{RF}$ to develop a substantial effect, one has to wait for time *T*, which satisfies the condition $T\omega_{RF}e_1 > 1$, where $e_1 = \left|\overline{e^{i\Phi_k(t)}}\right|$ is the average of the exponential phase factor in Eq. (8) over one MAS period. This involves many phase multipliers ($\omega_{MAS}/(\omega_{RF}e_1)$), which eventually average to 0 unless $\frac{\Omega}{\omega_{MAS}}$ is an integer. In turn, this leads to the resonance condition $|\Omega| = \frac{n}{2}\omega_{MAS}$ with an even *n* in the absence of motions. It is possible to estimate the width of the resonance $\delta\Omega$ as $\frac{\delta\Omega}{\omega_{MAS}}\frac{\omega_{MAS}}{\omega_{RF}e_1}\sim 1$, which leads to $\delta\Omega \sim \omega_{RF}e_1$.

In second-order perturbation theory,

$$a_k^{(2)}(t) = \omega_{RF}^2\sum_l \delta L_{kl}\delta L_{l,7}\int_0^t dt_2\, e^{-i\Phi_k(t_2)+i\Phi_l(t_2)}\int_0^{t_2} e^{-i\Phi_l(t_1)}dt_1 \quad \text{(Eq. S4)}$$

Non-zero values of $a_k^{(2)}$ are possible for *k* = 5,..., 8. We consider them separately. For *k* = 5, $\hat{K}_+$ coherence, there are non-zero terms with *l* = 1 and *l* = 4 for which $\delta L_{5,1}\delta L_{1,7} = -1/2\sqrt{2}$ and $\delta L_{5,4}\delta L_{4,7} = 1/2\sqrt{2}$, respectively. The integrals over time are

$$\int_0^t dt_2\, e^{-i\Omega t_2 \pm i\Phi_0(t_2)}\int_0^{t_2} e^{-i\Omega t_1 \mp i\Phi_0(t_1)}dt_1. \quad \text{(Eq. S5)}$$

Thus again, we see that outside the integer rotary resonances conditions, the mismatch between the periodicity of $\Phi_0(t)$ and aperiodicity of Ωt leads to the suppression of the transition amplitude. Without the suppression, the $a_k^{(2)}(t)$ amplitude would be $\sim\omega_{RF}^2 t^2$ and, if both integrals over t_1 and t_2 are suppressed, $a_k^{(2)}\sim\frac{\omega_{RF}^2}{\omega_{MAS}^2}$. However, there is an intermediate level of suppression in the case of the half-integer rotary resonances given by the odd values of *n* in the $|\Omega| = \frac{n}{2}\omega_{MAS}$ condition. For such offsets Ω,

the phase mismatch in the integral over t_1 is compensated by the opposite phase mismatch in the integral over t_2. This condition is more obvious if one makes the substitution $t_1 \rightarrow t_2 - t_1$. The double integral is then $\int_0^t dt_2\, e^{-2i\Omega t_2 \pm i\Phi_0(t_2)} \int_0^{t_2} e^{i\Omega t_1 \mp i\Phi_0(t_2 - t_1)} dt_1$. Further, there is an uncompensated phase change during a single MAS period only in the integral over t_1. Therefore, for the half-integer resonances, the amplitude is $a_k^{(2)} \sim \frac{\omega_{RF}^2}{\omega_{MAS}} t$, leading to a much less pronounced effect than for the integer resonances. In analogy with the estimate of the width of the integer rotary resonance above, we can conclude that the half-integer resonance width should be of the order of $\delta\Omega \sim \frac{\omega_{RF}^2}{\omega_{MAS}} e_1^2$. The value of $e_1 = \left|\overline{e^{\iota\Phi_k(t)}}\right|$ in this case is different from that in the full resonance case because of the different value of Ω when the phase factor of Eq. (S4) is considered.

For $k = 6$, $\hat{K}_-$ coherence, this case is similar to the k=5 case with the appropriate changes of signs in the exponents of Eq. (S5) and with $l = 2, 3$ in Eq. (S4).

For $k = 7, 8$ coherences $\hat{S}_z$ and $\hat{Q}$ in Eq. (S4), there are four possible intermediate states with $l = 1,.., 4$. The time integral for $l = 1$ is
$\int_0^t dt_2\, e^{i\Omega t_2 + i\Phi_0(t_2)} \int_0^{t_2} e^{-i\Omega t_1 - i\Phi_0(t_1)} dt_1$. Similar expressions hold for the other values of l. Both integrals are suppressed outside the integer resonance condition.

B) Rationalizing the relaxation trends of Figure 5 based on the second-order perturbation theory framework

Using the theoretical description of part A), we can rationalize the trends observed for the R_{CEST} rates shown in Figure 5 of the main text and described in the main text by considering the side-band intensities created by MAS. Effective relaxation is caused by the redistribution of intensities among the side bands. For low values of C_q, high frequency MAS does not create significant side-band intensity, preventing efficient relaxation. For high values of C_q, both high and small MAS frequencies create significant side-band intensity and the efficiency of relaxation thus depends on whether the side band closest to the chosen $\Omega/2\pi$ value has larger or smaller intensity. For the central band, these conditions imply that the most effective relaxation is in the vicinity of $C_q \sim \omega_{MAS}/2\pi$. The dependence of R_{CEST} on ω_{RF} is quadratic when $\omega_{RF} < |\Omega|, \omega_{MAS}$, as can be seen from Eq. (S3). Coherent evolution leads to $a_k^{(1)}(t) \sim \omega_{RF}$ for single-quantum coherences, reducing the coefficient of the $\hat{S}_z$ coherence by a term $\sim\omega_{RF}^2$ (the sum of the squares of the coefficients is not altered by the coherent evolution). Conformational exchange effectively quenches single-quantum coherences by modulating the ω_Q values and thus irreversibly reduces the coefficient of the $\hat{S}_z$ term (detected in the CEST) by an amount proportional to ω_{RF}^2.

Table S1. Additional experimental and processing parameters for the spectral data in Figure 2.

Sample, spinning	*Number of scans*	*Exponential line broadening, Hz*
DMS, static	32	500
DMS, 10 kHz MAS	32	50
DMS, 25 kHz MAS	32	50
DMS, 60 kHz MAS	10	10
Fibrils, static	4096	500
Fibrils, 10 kHz MAS	1024	100
Fibrils, 25 kHz MAS	1024	100

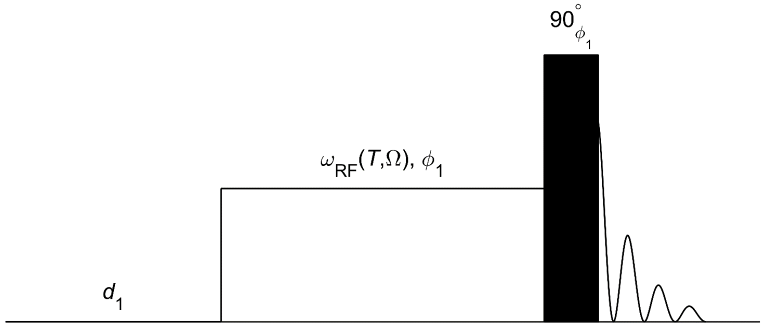

Figure S1. ^{2}H CEST pulse sequence under MAS. After the recycle delay d_1, a low-amplitude saturation pulse $\omega_{RF}(T,\Omega)$ is followed by a non-selective 90° pulse. ϕ_1= *x*, -*x*, receiver=*y*,-*y*.

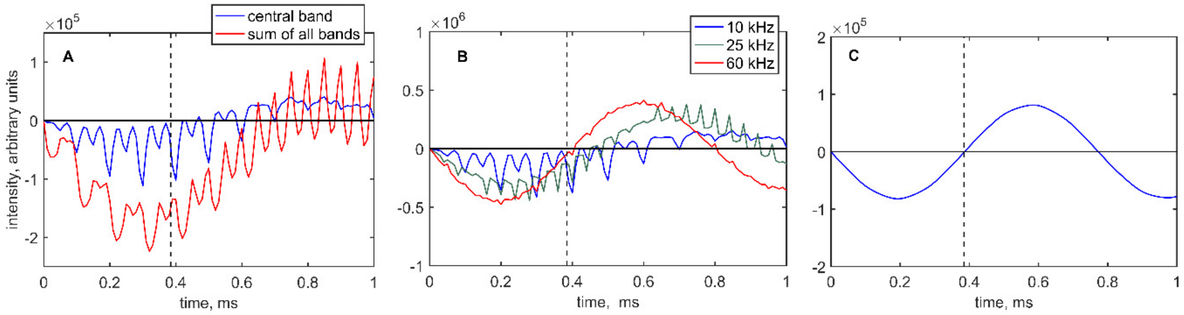

Figure S2. Simulated ^{2}H nutation experiment for the 1.3 kHz RF field and A) DMS effective tensor parameters (C_q=55.3 kHz, η=0) that include and 10 kHz MAS for the central band and the sum of all the bands. B) DMS tensor parameters and three MAS rates for the central band. C) A2 with the effective tensor for the free state in the presence of the diffusion, C_q= 3kHz and η=1, for 10 kHz MAS and the central band. The dotted lines show the positions of π/ω_{RF}, which is the expected position of the maxima in the absence of the quadrupolar interaction evolution during the nutation pulse.

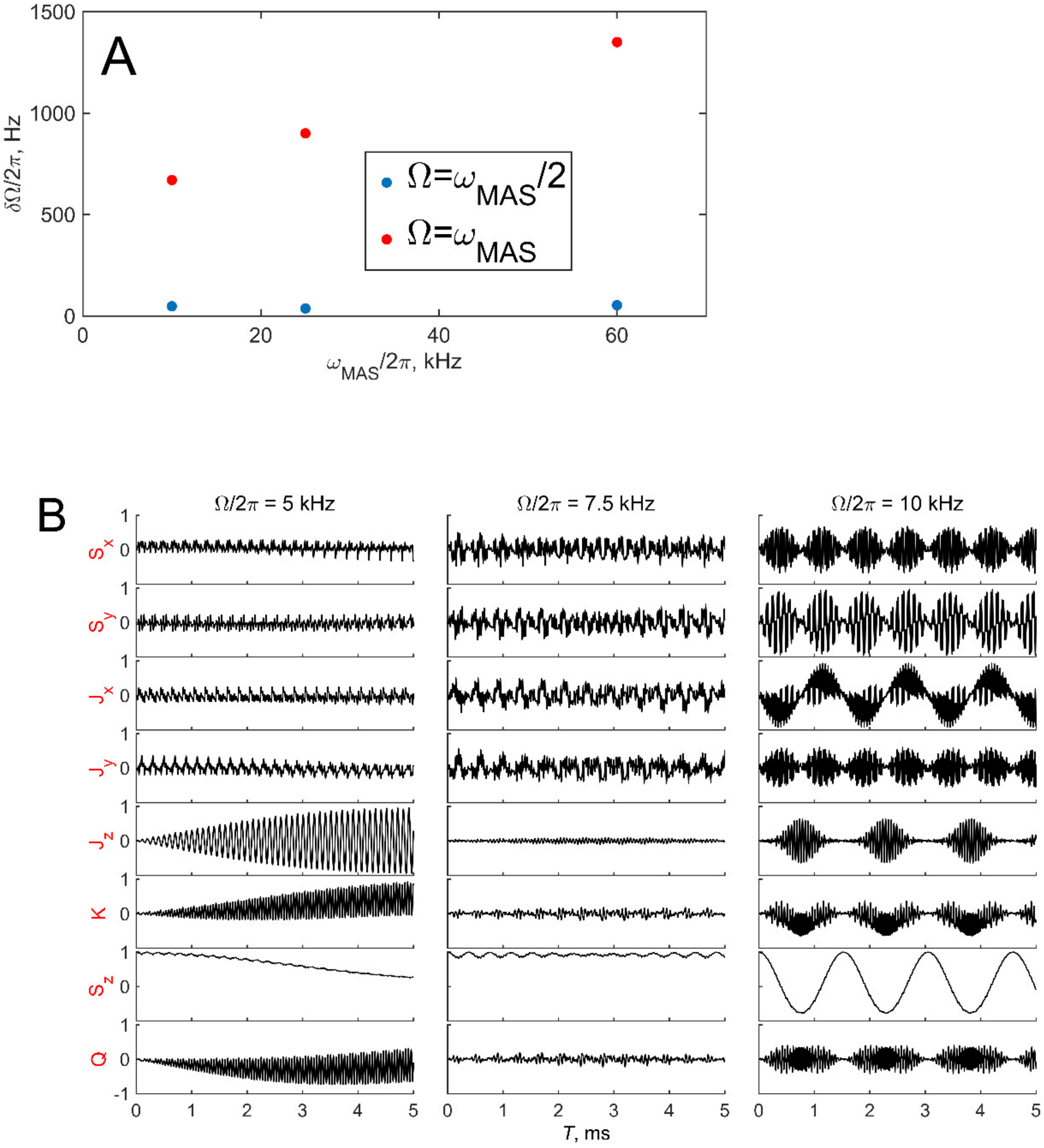

Figure S3. A) The widths of the coherent resonances: the full width at the half-depth for the first set of resonances at either $\Omega = \omega_{MAS}$ or $\Omega = \frac{\omega_{MAS}}{2}$ for three values of ω_{MAS}, with the tensor given by C_q=180 and η =0, calculated from the time evolution of the $\hat{S}_z$ coherence using Eq. (6). For every value of Ω, the depth is defined as the powder-average value of S_z averaged over 100 ms of evolution. B) The coherent evolution of the individual coherence in the CEST simulations for a single crystallite corresponding to the 30° orientation with respect to the magic angle for three values of $\Omega/2\pi$. $\omega_{RF}/2\pi$ = 1.3 kHz, $\frac{\omega_{MAS}}{2\pi}$ = 10 kHz, C_q= 55.3 kHz, and η = 0. These coherences remain real at all times; however, they are not eigenfunctions of L_0 in Eq. (S1).

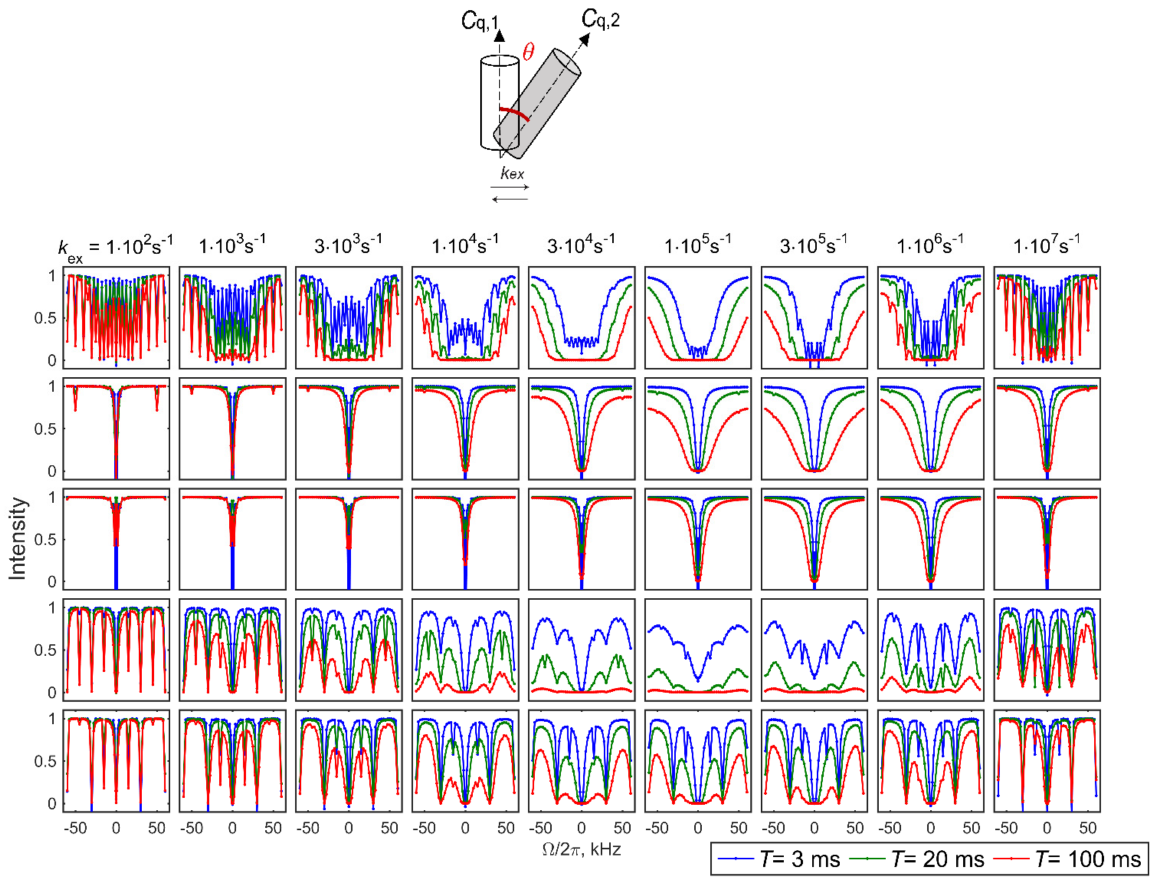

Figure S4. Simulated ^{2}H CEST profiles for the 2-site jump models for two axially symmetric tensors with different motional parameters and MAS rates for $\omega_{RF}/2\pi$ =1.3 kHz, and three saturation times T. The integrated intensity of all the spectral side bands normalized to the intensity at T=0 versus $\Omega/2\pi$. The offsets were sampled using a 2.5 kHz grid, which may affect the visual width of the half-integer rotary resonances in some cases.

A) $C_{q,1}$=$C_{q,2}$=55 kHz, θ=106°, p_1=50%, $\omega_{\mathrm{MAS}}/2\pi$ = 10 kHz.

B) $C_{q,1}$=$C_{q,2}$=55 kHz, θ=106°, p_1=50%, $\omega_{\mathrm{MAS}}/2\pi$ = 100 kHz.

C) $C_{q,1}$=$C_{q,2}$=20 kHz, θ=106°, p_1=50%,$\omega_{\mathrm{MAS}}/2\pi$ = 120 kHz.
This is the limit of $C_q \ll \omega_{\mathrm{MAS}}/2\pi$.

D) $C_{q,1}$=20 kHz, $C_{q,2}$=180 kHz, θ=0°, p_1=50%, $\omega_{\mathrm{MAS}}/2\pi$ = 30 kHz.

E) $C_{q,1}$=$C_{q,2}$=55 kHz, θ=106°, p_1=10%, $\omega_{\mathrm{MAS}}/2\pi$ = 30 kHz.

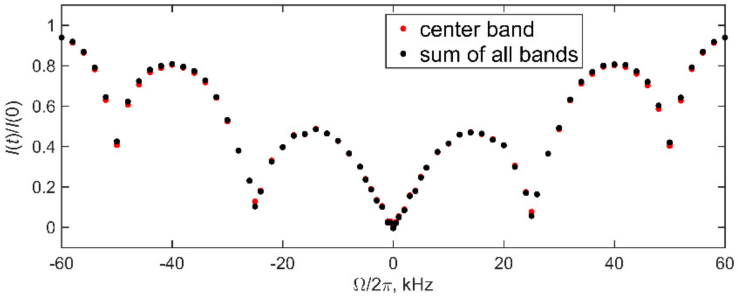

Figure S5. Comparison of the experimental ^{2}H CEST profiles for the central band and sum of the five bands (shown in the spectra of Figure 2 of the main text) for DMS at the 25 kHz MAS rate, RF field strength of 1.3 kHz, and T = 20 ms, collected at 17.6 T and 76°C.

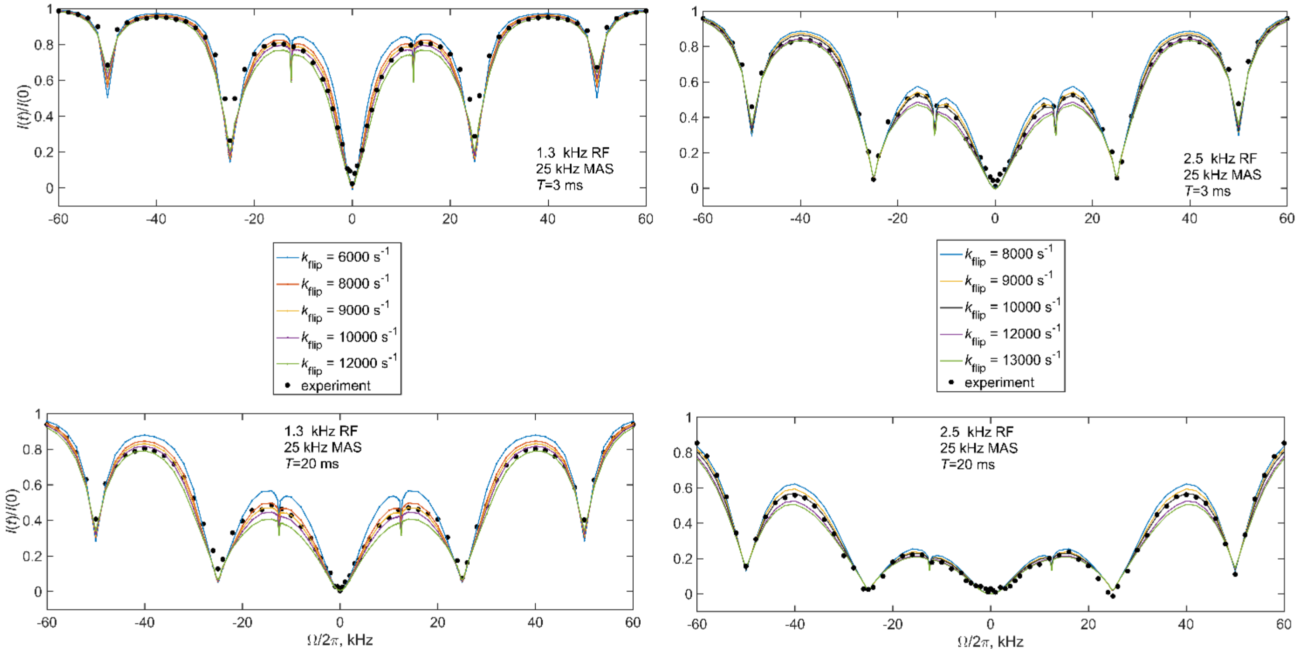

Figure S6. Sensitivity of the CEST profiles to the flip rate in DMS at the 25 kHz MAS rate. Simulated profiles for several values of the k_{flip} rate constant are overlaid with the experimental data. The values of the RF fields, saturation times, and k_{flip} rate constants are shown directly on the panels.

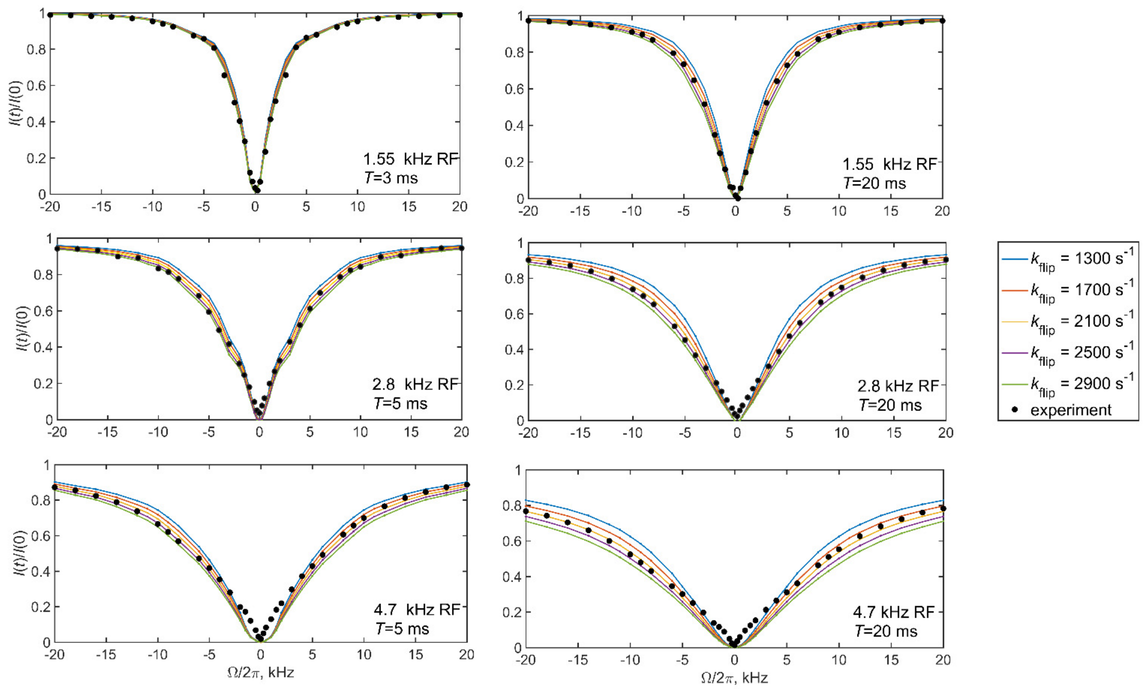

Figure S7. Sensitivity of the CEST profiles to the flip rate in DMS at the 60 kHz MAS rate. Simulated profiles for several values of the k_{flip} rate constant are overlaid with the experimental data. The values of the RF fields, saturation times, and k_{flip} rate constants are shown directly on the panels.

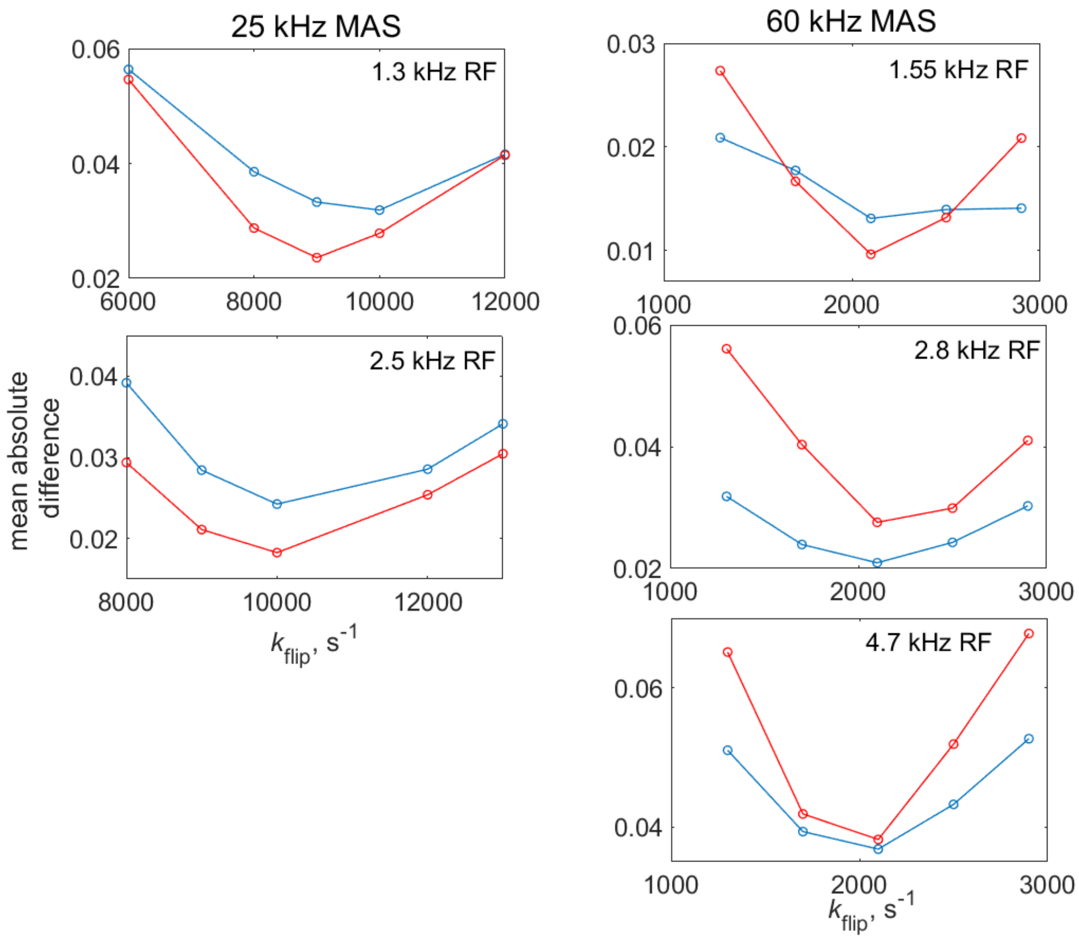

Figure S8. Mean absolute difference between the experimental and simulated CEST profiles for DMS in Figures S5 and S6. The values of the RF fields are indicated directly on the panels. The blue symbols represent $T = 3$ ms and red symbols represent $T = 20$ ms.

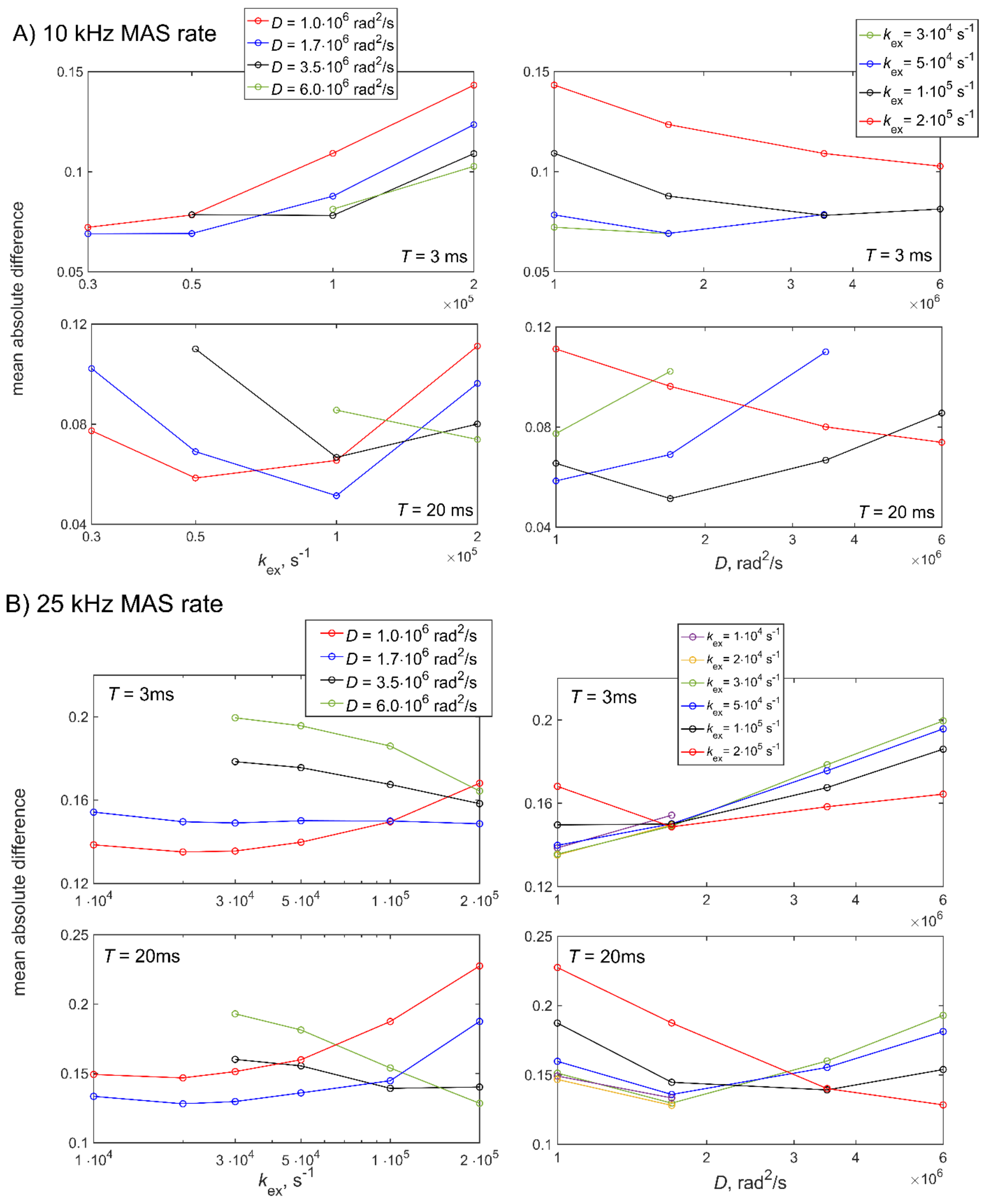

Figure S9. Mean absolute difference between selected offsets of the experimental and simulated ^{2}H CEST profiles of the A$\beta_{1\text{-}40}$ fibrils (labeled at the A2-CD_3 side chain) at a 1.3 kHz RF field strength and saturation times shown directly on the panels for the 10 kHz MAS rate (A) and 25 kHz MAS rate (B). The offsets are (±2, ±4, ±6, ±8, ±14, ±16, ±24, ±26 and ±10, ±20, ±30 kHz) for 1the 0 kHz MAS rate and (±2, ±4, ±6, ±8, ±10, ±16, ±18, ±20, and ±12.5, ±25, ±50 kHz) for the 25 kHz MAS rate. The left column shows the dependence on the value of *D* for several values of k_{ex}, while the right column shows the dependence on k_{ex} for several values of *D*.

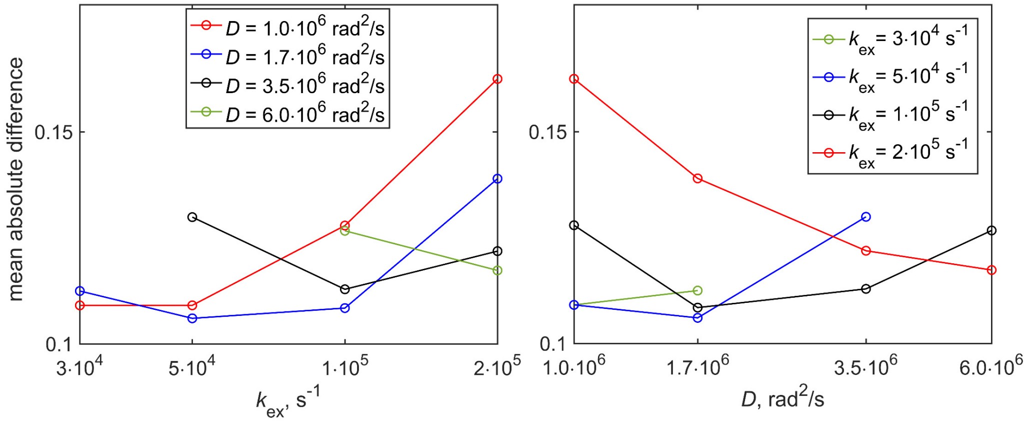

Figure S10. Combined mean absolute difference between selected offsets of the experimental and simulated ^{2}H CEST profiles of the A$\beta_{1\text{-}40}$ fibrils for all of the experimental conditions, listed in Figure S9.

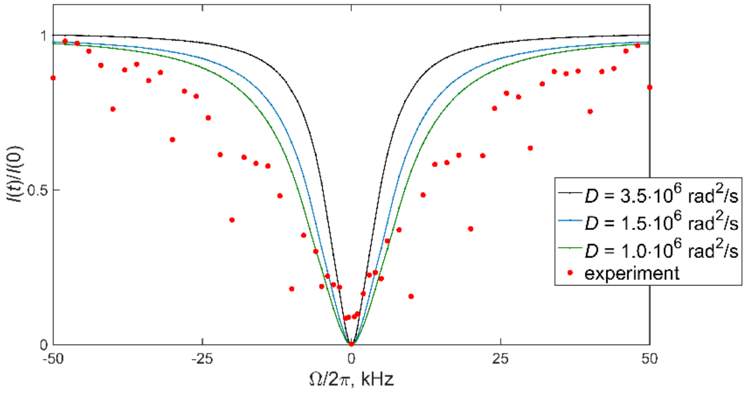

Figure S11. Comparison of the experimental normalized ^{2}H CEST profiles for the A2-CD_3 Aβ fibrils at the 10 kHz MAS rate, 1.3 kHz RF saturation field strength, and T= 20 ms with the simulations including the diffusion of the free state in the absence of the conformational exchange between the free and bound states. The chosen values of D are shown directly on the graph.

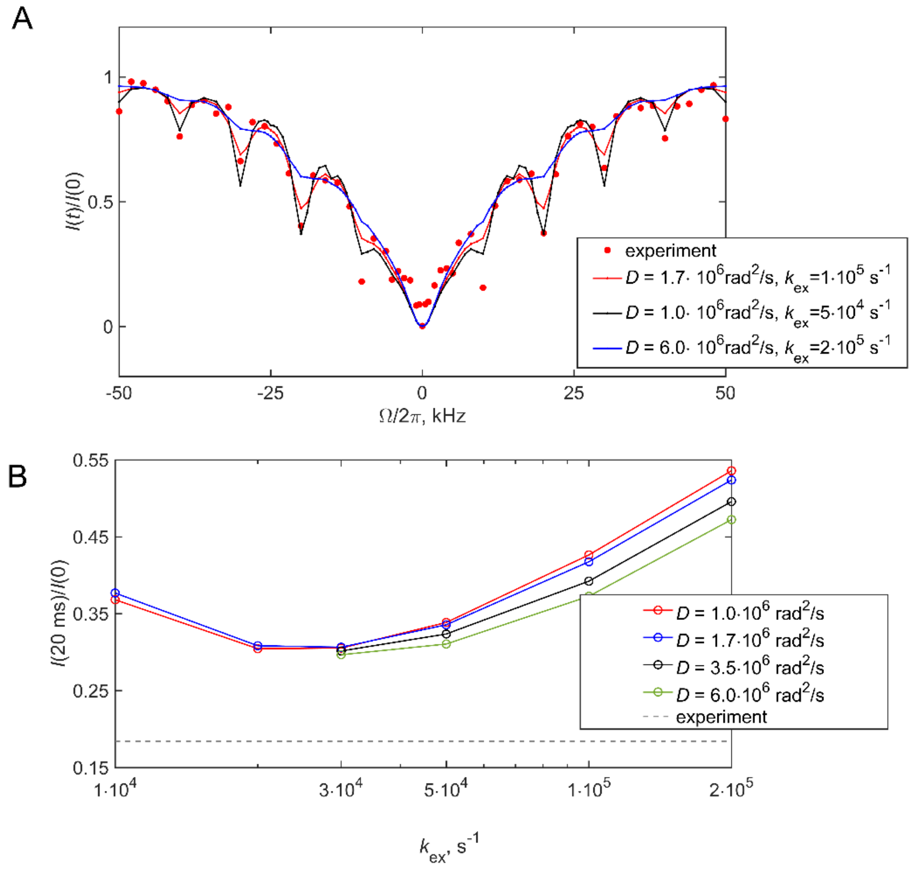

Figure S12. A) Alternative fits of the experimental ^{2}H CEST profiles of the A2-CD_3 A$\beta_{1\text{-}40}$ fibrils at the 10 kHz MAS rate, $\omega_{RF}/2\pi$ =1.3 kHz, and T = 20 ms for different sets of the D and k_{ex} parameters with relatively low values of the mean absolute difference, as shown in Figure S8. B) The simulated ^{2}H CEST intensities I(20 ms)/I(0) for the offsets corresponding to the first integer resonances, $\Omega/2\pi$ = ±25 kHz, at the 25 kHz MAS rate and $\omega_{RF}/2\pi$ =1.3 kHz as a function of k_{ex} for different fixed values of D. The dotted line shows the average experimental value I(20 ms)/I(0) for these two offsets.

References

Bain, A.D., and Berno, B. (2011). Liouvillians in NMR: the direct method revisited. *Prog. Nucl. Magn. Reson. Spectrosc.* 59**,** 223-244.

Brinkmann, A. (2016). Introduction to average Hamiltonian theory. I. Basics. *Concepts Magn. Reson. A* 45A**,** e21414.

Haeberlen, U., and Waugh, J.S. (1968). Coherent averaging effects in magnetic rpesonance. *Phys. Rev.* 175**,** 453-467.
